# The Human Frontal Lobes and Frontal Network Systems: An Evolutionary, Clinical, and Treatment Perspective

**DOI:** 10.1155/2013/892459

**Published:** 2013-03-14

**Authors:** Michael Hoffmann

**Affiliations:** ^1^Director Stroke and Cognitive Neurology Programs, James A. Haley Veterans' Hospital, 13000 Bruce B. Down's Boulevard, Tampa, FL 33612, USA; ^2^Cognitive Neurologist and Director SciBrain, Roskamp Neurosciences Institute, 2040 Whitfield Avenue, Sarasota, FL 34243, USA

## Abstract

Frontal lobe syndromes, better termed as frontal network systems, are relatively unique in that they may manifest from almost any brain region, due to their widespread connectivity. The understandings of the manifold expressions seen clinically are helped by considering evolutionary origins, the contribution of the state-dependent ascending monoaminergic neurotransmitter systems, and cerebral connectivity. Hence, the so-called networktopathies may be a better term for the syndromes encountered clinically. An increasing array of metric tests are becoming available that complement that long standing history of qualitative bedside assessments pioneered by Alexander Luria, for example. An understanding of the vast panoply of frontal systems' syndromes has been pivotal in understanding and diagnosing the most common dementia syndrome under the age of 60, for example, frontotemporal lobe degeneration. New treatment options are also progressively becoming available, with recent evidence of dopaminergic augmentation, for example, being helpful in traumatic brain injury. The latter include not only psychopharmacological options but also device-based therapies including mirror visual feedback therapy.

## 1. Definition and Synonyms

Clinically, frontal lobe syndromes, frontal network syndromes, frontal systems syndromes, executive dysfunction, and metacognition have all been used to describe disorders of frontal lobes and their extended networks although they are not all synonymous. Anatomically they refer to those parts of the brain rostral to the central sulcus. However, because the frontal lobes network with every other part of the brain, strictly speaking, frontal network syndromes constitute the most accurate neurobiological depiction. The term, frontal network syndromes (FNS,) emphasizes the universal connectivity of the frontal lobes with all other brain regions. For example, the stroke literature is replete with FNS that have been reported with discreet lesions outside the anatomical boundary of the frontal lobe, such as subcortical grey matter, subcortical white matter, with isolated lesions of the brainstem, cerebellum, temporal, and parietal lobes [1–8].

**Figure 1:**
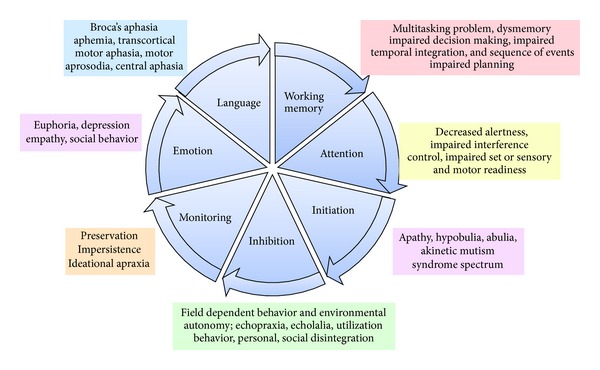
Proposed core frontal systems.

For the purposes of simplification, five primary syndromes and numerous secondary syndromes may be delineated. Impairment in working memory, executive function, abulia, disinhibition, and emotional dyscontrol may be regarded as the elementary deficits of FNS. In addition, a number of secondary manifestations may be identified such as a wide array of behavioral abnormalities such as loss of social norms, imitation behavior, compulsions, and obsessions [9, 10] (Figure 1).

## 2. Evolutionary Aspects and Relevance to Clinical Syndromes

To begin to understand the most complex object in the universe, the human brain and in particular the frontal lobes, it is most illuminating to study the evolution of our mind and thereby gain a better understanding of the clinical syndromes we are faced with today. In the words of Theodosius Dobzhansky, “nothing in biology makes sense except in the light of evolution” [11]. Life on earth evolved approximately 3.7 billion years ago and thereafter continuously shaped by extra-terrestrial and geological events, punctuated by a number of key events. The inclusion of prokaryotes into eukaryotic cells furnished cells with a powerhouse, the mitochondria. Some time after “Snowball Earth,” when glaciers reached the equatorial regions about 620–590 million years ago (mya), with the Cambrian explosion of organism diversity, vertebrates (bony fish, amphibians, reptiles, birds, and mammals) formed (~520 mya) [12]. Formation of the vertebrate skeleton allowed rapid movement, an advanced nervous system, and high degree of encephalization even though 98% of animal species are invertebrates versus 2% being vertebrate. Myelination enabled a vastly improved neural transmission, speeding up neural transmission by a factor of 10 (~9 meters per second in unmyelinated fiber versus 50–100 meters per second in myelinated fiber), with increased temporal precision, faster communication between the brain and body parts, and ability to react more rapidly to prey and predator [13, 14]. With warming conditions, fish evolved lungs and walked on land about 365 mya, mammalian evolution (~200 mya) and subsequent proliferation after dinosaur extinction (~65 mya). Mammals developed advantageous thermoregulation, thanks to fur and the advantage of mammalian glands [12]. Primates evolved about 85 mya and about 6 mya the “East Side Story” event (African Rift Valley formation leading to a hot and dry East Africa) precipitated bipedalism, increase in brain size, and tool making. The emergence of dopamine as a key neurotransmitter was critical in cooling our bodies and brains in a thermally stressed environment and later exapted for executive function [15]. Around this time, our frugivorous diet (since ~60 mya) was supplemented with meat and with the advent of Marine Isotope Stage 6 (180–120 mya) that may have also served as an important event that highlighted the key dietary changes to sea-food that may have played a factor in advancing our cerebral connectivity that ultimately made us modern humans [16, 17]. Shell fish (scallops oysters and prawns) are rich in both iodine and essential fatty acids, both of which have been correlated with boosting dopamine activity and intellectual development [18]. Morphological brain changes as well as connectivity changes were key features in our development.

### 2.1. Brain Volume

As a starting point using the so-called “missing link” hominid, *Australopithecus africanus* (brain volume approximately 450 mL), there was an increase in size to approximately 1500 mL in Neanderthals over a 3 million year period and subsequently a slight decrease again in modern humans *Homo sapiens sapiens* to 1350 mL [37]. During this time, there was a reduction in the size of the visual striate area (BA 17) with a relative increase in the posterior parietal cortices and frontal lobe reorganization at the network, neurotransmitter, and receptor levels [38].

### 2.2. Frontal Lobe Size

The size of frontal lobes in various mammalian species is frequently cited as steadily progressing allometrically from the so-called lower forms (rats and mice) to dogs and cats with primates and humans having the biggest proportionally. The frontal lobes comprise of 37%–39% of the cerebral cortex macroscopically and connect to all other parts of the brain, often in a reciprocal manner [39]. The frontal lobe in humans is as large as that would be calculated for an ape of human brain size overall, not larger as is often reported [40]. However, what sets us apart from other mammals is not so much brain size but reorganization of our brains in terms of connectivity and neurotransmitter changes. These changes may be summarized in the following manner.

1. Progressive increase in size.
2. Hemispheric asymmetry, also called cerebral torque (right frontal and left occipital petalias).
3. Neuropil reorganization.
4. Reorganization in terms of neurotransmitter systems.
5. Receptor modification [41].

### 2.3. Histological Architectural Changes of the Neuropil 

#### 2.3.1. Frontal Lobes

Axons, dendrites, and space between the neurons and glial cells constitute the neuropil which is decreased to BA 10 in humans relative to other primates. In Broca's area (BA 44, 45), the cortical architectural units or minicolumns are wider in humans relative to primates. BA 10 constitutes the frontopolar and BA 13 the posterior fronto-orbital region. BA 10 is twice as large in terms of overall brain volume compared to any of the other great apes (1.2% in humans versus 0.46%–0.74% in great apes). Interestingly, BA 13 is relatively reduced in humans [42].

Spindle cells or von Economo cells appear in layer Vb in both the fronto-insular cortex as well as the anterior cingulate cortex, only in humans and African (not Asian) great apes and are approximately 30% more numerous in the right hemisphere of these species [42]. As they arose in our last common ancestor about 10 mya, they probably subserve the role of social and emotional processing which arose many millions of years before language [43]. In view of this, they may constitute one of the neurobiological deficits of autism. As an example of convergent evolution, other intelligent species such as the cetaceans have spindle cells [44].

The neuronal density of the important BA 10 and 13 in humans is about half (human BA 10~32000 and BA 13~30000 neurons per cubic millimeters) that of the great apes (chimpanzee BA 10~60000 and BA 13~43000) and is often much less than half (orangutan BA 10~78000 neurons per cubic millimeter). The increase in neuropil particularly of BA 10 is likely related to the connectivity of this region with other tertiary association cortex and the other hemisphere [45]. 

#### 2.3.2. The Temporal Lobe

Surprisingly, this cortical region is larger in size in humans than would be predicted for an ape of human brain size. There is a relative increase in the size of white matter, and the ratio of gyral-to-core white matter in the temporal lobes is larger than would be predicated for other hominoids. This relatively enlarged gyral white matter compared to core white matter is interpreted as reflecting greatly increased interconnectivity subserved by short association fibers [46].

#### 2.3.3. The Amygdala

Of the component amygdaloid nuclei (lateral, basal, and accessory nuclei), the lateral nucleus is relatively larger in humans than would be expected in an ape of human brain size. This has been attributed to the increased interconnectivity with the temporal lobe's unimodal and polymodal sensory information [47]. 

### 2.4. Overall Brain Reorganization and Mosaic Systems

In human evolution, there has been a differential expansion and reorganization not only of the temporal lobes and amygdaloid complex, but also of the inferior parietal lobes. Specific networks evolved in a coordinated manner that has been termed mosaic evolution. This implies that evolution may have acted on neural systems rather than discrete anatomical structures [48]. Areas that are critical to social behavior which include the amygdaloid nuclei and limbic component of the frontal cortex are both volumetrically larger and revealed reorganization in their networks [42]. This is in direct contrast to the traditional view that limbic structures are conserved whereas the frontal lobes had enlarged. Within the frontal lobes themselves, however, many organizational and network changes have of course taken place. Structures and networks that are implicated in social and emotional processing include the orbitofrontal cortex, the amygdala, fronto-insular cortex, and temporal polar cortex with the latter also important in language processing [49]. These represent the so-called mosaic reorganization that has been a feature of human evolution [50].

### 2.5. Neurotransmitter Systems—Evolutionary Aspects

The quick acting excitatory (glutamate) and inhibitory (GABA) neurotransmitters (NT) act via ion channels with charged ions enabling a relatively quick response in terms of microseconds and seconds. The neuromodulators (NM) such as serotonin (5-HT), dopamine (DA), norepinephrine (NE), acetylcholine (Ach), and histamine (H) act differently in that they promulgate longer lasting and more diffuse actions via the G-protein cascade system. The ultimate outcome on a network may be excitatory, inhibitory activation of their own presynaptic autoreceptors or interaction synergistically with the other NM systems.

Overall, there is regional heterogeneity of neurotransmitters in the human brain. Different NT's subserve different higher cortical functions (HCF), and in neurodegenerative disease NT deficits occur in varying combinations. The downside of NT modulatory systems for intellectual advances probably made humans more susceptible to a number of neurodegenerative diseases, unique to humans [51].

#### 2.5.1. Dopamine (DA)

Within the frontal subcortical circuits, DA is the principal NT. The reasons for this have been proposed as part of a very plausible and well-researched hypothesis by Fred Previc and their open loop systems; DA and Ach became the predominant NT's in the left hemisphere and NE and 5HT in the right hemisphere [51]. 

DA is considered to have been one of the key factors in the emergence of human intelligence. After the geological events that led to the “East Side Story” with East African becoming relatively dry and arid, heat management and combating the deleterious effects of hyperthermia (including so called heat stroke) were a critical factor in survival of the mammals and the newly emerged bipedalist, Australopithecus africanus [15]. The function of DA in lowering body temperature presumably enabled early hominoids to better tolerate the hyperthermia of chase hunting and catching prey that succumbed to chase myopathy [52, 53]. Thereafter in an evolutionary sense, DA expansion occurred, due to increased calcium metabolism from prolonged aerobic activity as well as the increased tyrosine (a dopamine precursor) consequent to increasing meat supplementation about 2 mya [54]. The clinical sequelae of blocking dopamine (by drugs such as haloperidol, risperidone, and quetiapine) as malignant hyperthermia and neuroleptic malignant syndromes may be therefore interpreted in an evolutionary perspective. DA became exapted as the most important NT in our evolving brains, eventually concerned with most of the core frontal functions working memory, cognitive flexibility, motor planning, abstract representation, temporal sequencing, and generativity [51].

DA exerts a modulatory effect (affects signal-to-noise ratio) on the PFC G-protein linked receptors on dendritic shafts and spines of glutaminergic pyramidal neurons and dendrites of GABA-ergic neurons [55]. These neurobiological features enable DA to regulate working memory, reasoning, and language. Humans and great apes feature DA input to all cortical areas, in contradistinction to the paucity of DA-ergic innervation of rodents [56]. This was determined by measuring cortical DA innervation (axon density) using tyrosine hydroxylase immune reactivity. In addition, there is a regional DA-ergic distribution most intense in layer I and V-VI of the association cortices [57]. Furthermore, compared to great apes, humans have a generalized increased DA-ergic input to the prefrontal cortical regions. The dopaminergic hypothesis in human evolution purports that the expansion of human DA-ergic in particular was the most important factor in human tool making, exploration, cultural, and scientific developments [51]. This theory also proposes that the drawbacks are the propensity for hyperdopaminergic syndromes such as schizophrenia, bipolar disease, autism, attention deficit hyperactivity disorders, and neurodegenerative diseases.

#### 2.5.2. Serotonin

In humans and great apes, compared to other mammals, there is an overall increase in the cortical output 5-HT efferents [55]. The 14 different serotonergic G-protein-related receptors and one ion channel receptor (5-HT 3) enable the modulation of several different functions simultaneously, including memory, learning, and inhibition. This occurs via receptors on pyramidal cells and dendritic shafts and via interneurons which allows signal modulation from local circuits with reference to extrinsic stimuli [58]. From a clinical point of view, serotonin in the OFC circuitry has been linked to self-control, emotional, processing, and inhibition regulation [59]. 

#### 2.5.3. Acetylcholine

In the neuromodulatory axons of humans and great apes exist varicose type axons that are likely to have a role cortical plasticity [60], associated with advanced traits such as superior learning capability, social learning, advanced tool manufacture, and self-awareness. These effects are mediated via 5 muscarinic receptors (M1–M5), all of which are G-protein linked. Nicotinic receptors are all ligand gated ion channels. Both transmit mediating excitatory and inhibitory effects on GABA interneurons and pyramidal cells. The neurophysiological effects translate into cognitive flexibility, learning, and working memory [61].

#### 2.5.4. The Mosaic Cognitive Evolution

Hominoids are able to imitate behavior, and imitation ability of primates and hominoids was crucial to the cultural evolution. The imitation ability may be termed an “all purpose learning mechanism” [62]. The imitation circuitry likely evolved through anatomical, chemical, and organizational changes. The circuit has been termed “mosaic” in that it involves the PFC, parietal, temporal, and cerebellar regions. The core features of memory and attention as well as more specific cognitive domains such as language and tool use are presumed to be based on the mosaic pattern, itself based on the imitation behavior circuitry that may have a visual, auditory, and tactile dimension [62]. A Summary of cognitive psychological and neuroarcheological changes is depicted in Table 1.

## 3. Neurobiology: The Brain Is a Connectome Consisting of Both Neurochemical Tracts and Macroscopic Hard-Wired Tracts 

### 3.1. Neurochemical Tracts

The fast acting excitatory (glutaminergic) and inhibitory (GABA) amino acid neurotransmitters are modulated by a number of widely projecting slower acting (most G-protein linked) neurotransmitters. This type of chemical architecture is useful in coordinating many neurons and neuronal circuits in response to a stimulus or threat. There are currently 8 chemical or neuromodulatory tracts (DA, 5-HT, NE, Ach, H, oxytocin/vasopressin, and orexin) (Figure 3) with their nuclei of origins in the brainstem, basal forebrain or hypothalamus, and extensive cortical ramifications, two examples of which are depicted in Figures 3 and 4 [1, 2].

### 3.2. Network Neuroanatomy

#### 3.2.1. The Major Cerebral Fasciculi as They Pertain to the Frontal Lobes and Network Systems

The main functions of the frontal lobes are motor action and the temporal integration of behavior. Frontal lobe evolution may be seen as progressive refinement of pyramidal pathway responses (motor, speech, and behavior) by incorporating cognitive and emotional processes. Optimal decision-making requires a flexible system that can incorporate a wide range sensory inputs, at the same time prioritizing and choosing the most effective response in a changing environment. This has resulted in a complex circuitry. This is achieved through the major cerebral network systems: superior frontal occipital fasciculus, superior longitudinal fasciculus, inferior longitudinal fasciculus, cingulum and uncinate fasciculus, and seven frontal subcortical circuits [63, 64]. The major fasciculi are depicted in Figure 5 and can also be imaged by diffusion tensor imaging with directional specificity that is color-coded.

1. U fibers (orange).
2. Superior occipito-frontal fasciculus (royal blue).
3. Superior longitudinal fasciculus (pink).
4. Inferior longitudinal fasciculus (dark green).
5. Perpendicular fasciculus (dark blue).
6. Uncinate fasciculus (purple).
7. Arcuate fasciculus (light blue)—note two tracts.
8. Corpus callosum (brown).
9. Cingulum (red).
10. Fornix (light green).

#### 3.2.2. The 7 Frontal Subcortical Networks (FSC)

The FSC share a similar basic anatomy, namely, cortex-caudate-globus pallidus-thalamus cortex (Figures 6 and 7). Confusion of terminology may exist because of the rather cumbersome terminology related to subcortical structures, best delineated by a diagrammatic representation (Figure 8). The neurotransmitters and neuropeptides integrated in this circuitry are mainly excitatory (glutamate) and inhibitory (GABA) being the principal ones but many others involved in a neuromodulatory capacity including the monoamines, enkephalin, neurotensin, substance-P, dynorphin, adenosine, and neuropeptide-Y [9, 65–67].

#### 3.2.3. The FSC Connectivity

- Direct and indirect pathways.
- Connections with the other circuits (corticocortical).
- Connections to areas outside the FSC's.

1. *Direct Pathway*. Glutamate release occurs from frontal cortical regions to the striatum, mostly caudate nucleus, less often to putamen and nucleus accumbens. This releases GABA at internal segment of GP and SN; GABA from GPi (globus pallidus interna) to thalamus diminishes and in turn the thalamus increases glutaminergic excitation of cortical regions. The striatal neurons within this pathway that project to the GPi are termed striosomes with D1 receptors. The net result is a thalamic disinhibition [9, 65, 66].
2. The indirect pathway balances the direct pathway. Here the striatal efferents are termed matrix efferents wirt D2 receptors and project from the globus pallidus externa (as opposed to interna) with a net thalamic inhibition. Some of the GPe neurons are cholinergic but most are GABA-ergic with two different types of GABA ergic cell types within the GPe termed GP-TI and GP-TA, which mediate crosstalk between the afferent and efferent circuits of the GPe as well as the direct and indirect pathways [68].
3. Connections to regions outside the FSC's include (i) DLPFC (BA 46) to parietal (BA 7), (ii) anterior cingulate to temporal lobe (hippocampus, amygdala, entorhinal cortex), (iii) medial OFC to temporal lobe and iv) lateral OFC to heteromodal sensory cortex [9, 69]

There is a complex interplay of the neurotransmitters, receptors, and circuit plasticity such as enhancement of striatal dopamine release by cholinergic agonists. The importance of recognizing and understanding this circuitry is in the relationship they have to clinical syndromes clinicians appreciate with regard to frontal pathology. These may be conveniently conceived of as three principal syndromes of the following.

1. Predominant dorsolateral prefrontal cortex (DLPFC): temporal organization of information, executive function, working memory, and multitasking [70].
2. Anterior cingulate circuitry: motivation for behavior. Impairments here lead to akinetic mutism and the abulic spectrum of disorders [71].
3. Orbitofrontal (OFC) circuitry: the cortical component of the limbic system where the emotional and other limbic components are integrated into behavioral output. Hence, disinhibitory syndromes may occur in response to lesions of the medial as well as the lateral OFC [9].
  - The medial OFC circuitry mediates empathic and socially appropriate behavior. Personality change in the context of frequently normal cognitive (DLPFC) function is usual and a manifold of presentations is encountered principally. The various forms of echopraxia or imitation behavior, utilization behavior, and environmental dependency syndromes (field-dependent behaviors) are sometimes the overriding clinical manifestations of these lesions. From a neurophysiological point of view, the medial OFC attaches emotional valence to events in turn determines the strength of the episodic memory. Impaired autonomic and endocrine processing is associated with lesions of the medial OFC [72].
  - Lateral OFC lesions have been correlated with OCD, depression, irritability, mood disorders, and field-dependent behaviors. Obsessive compulsive disorders spectrum group of disorders include OCD spectrum itself that comprises of obsessions (intrusive urges thoughts, images), compulsions (repetitive, ritualistic type of activities of a physical or cognitive nature), Tourette's syndrome, kleptomania, risk seeking behavior, pathological gambling, body dysmorphic disorder, and the immune disorder of pediatric autoimmune neuropsychiatric disorders (PANDAS) [73, 74].

In addition to these three principal behavioral FSC's, two others have recently been added. The inferotemporal subcortical circuit may be associated with deficits in visual discrimination, visual scanning, visual hallucinations, and psychosis [65]. In addition, a circuit between the posterior parietal region (BA 7) and the prefrontal region (BA 46) also contributes to processing of visual stimuli of significance and accordingly visuospatial processing [69].

**Figure 2:**
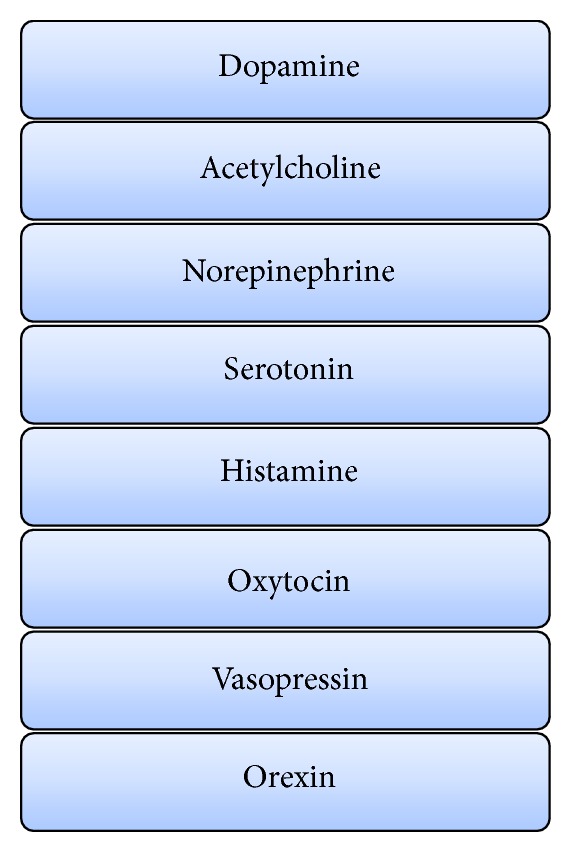
The 8 modulatory systems

**Figure 3:**
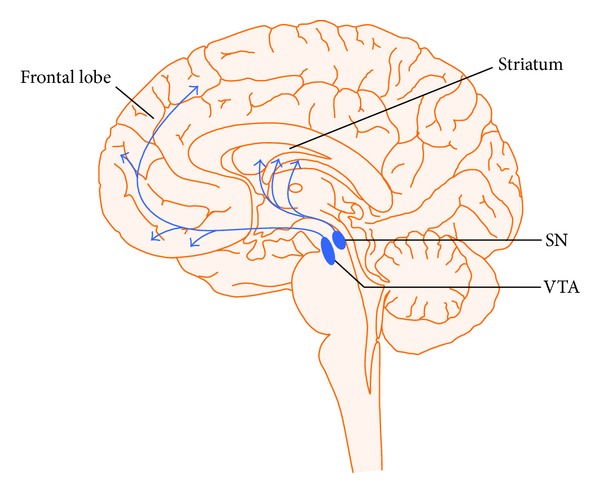
Dopamine system.

**Figure 4:**
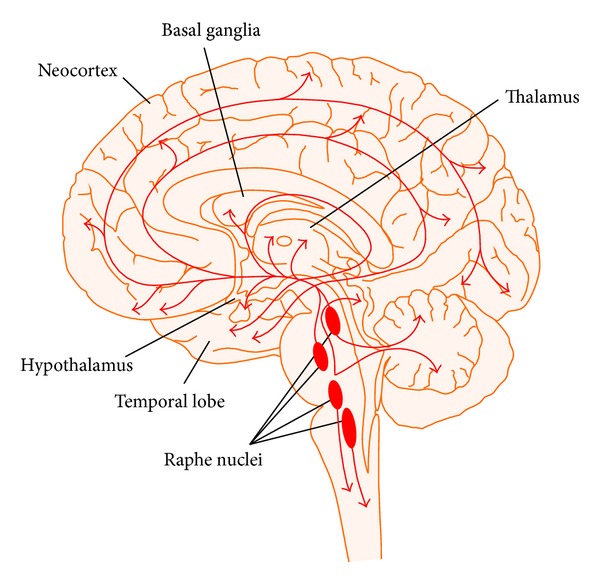
Serotonin system.

**Figure 5:**
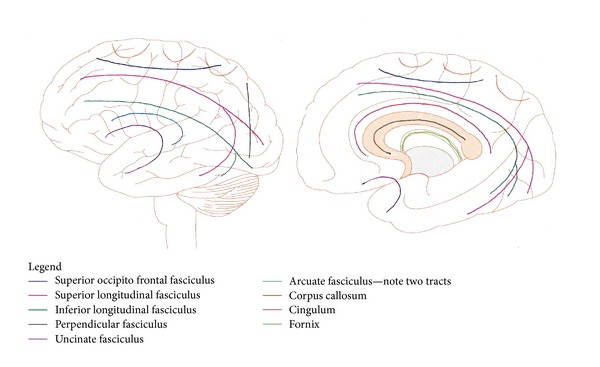
The major fiber tracts of the brain, lateral and medial.

**Figure 6:**
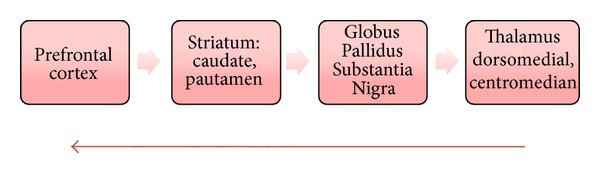
Frontal subcortical circuits. Parallel circuits each with the same components. Exception; medial PFC via n. accumbens instead of caudate nucleus.

**Figure 7:**
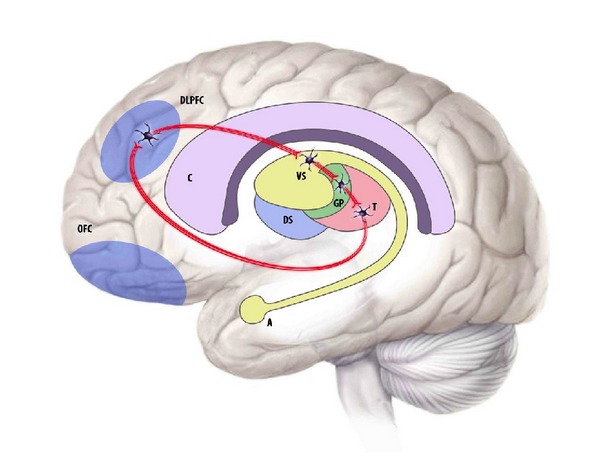
Dorsolateral prefrontal executive frontal subcortical circuit.

**Figure 8:**
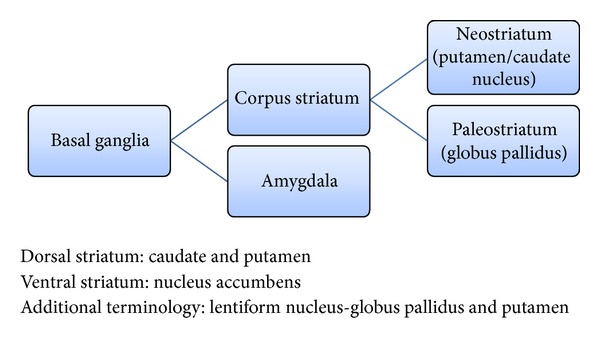
Basal ganglia anatomy.

Individual lesions or disease processes usually affect more than one to differing degrees and in various combinations. To facilitate the diagnostic components and sometimes treatment options, it is useful to consider the FSC's and their clinical counterparts in terms of neurophysiological core components and in correlation to the clinical syndromes (Figure 2).

## 4. Clinical: Recognizing Primary and ****Secondary Syndromes of FNS and Their Usefulness in Treatment Strategies

From a clinical point of view, a vast panoply of symptoms have been associated with frontal lobe lesions but may conveniently grouped under the following primary domains.

*A. Primary (Figure 2)*. These include initiation, disinhibition, working memory, attention, monitoring, language, and emotional control.

*B. Secondary (Figure 2)*. Phenotypic presentations of these primary processes may include the following.

*Initiation*. The hypobulia, abulia, pathy, and akinetic mutism spectrum of disorders. In addition, loss of creativity, curiosity, and initiative may also be present

*Disinhibition*. Impulsivity: field-dependent behavior forms and manifestations. These include (UB, IB, EDS) loss of judgment, loss of insight, impairment comportment, inappropriate social behavior, loss of empathy, irritability, aggression, irascible, excessive jocularity, irresponsible behavior, restlessness, hyperactivity, hypersexuality, hyperorality, and incontinence.

*Working Memory*. This include verbal and nonverbal working memory, multitasking, abstract thought, planning ahead, temporal sequence of events. Examples of executive functions include learning new information both verbal and visuospatial, searching memory systems, activation of past memories, temporal organization of behavior, attention, and generation of motor activity that includes speech, writing, or limb movement. These involve at least four of the five subprocesses: task setting, initiation of the task, monitoring, error detection, and behavioral self-regulation [75]. These are, therefore, modulated by up to four of the FSC's with task setting correlating with left DLPFC and monitoring/error detection correlating with right DLPFC activity. Initiation of a new task involves both left and right superior medial frontal FSC's and behavioral regulation disrupted after medial OFC circuit lesions [76]. Cerebral lesions distinct from the FSC's may be associated with FNS such as parietal lesions [9]. Even memory analysis can at times be correlated with FSC topography. For example, caudate lesion patients may have poor recall but relative preservation of recognition. Thalamic lesion patients on the other hand may have impairment of recall and recognition [77]. 

*Attention*. Under this rubric are included alertness and arousal. Regarding differentiating attention and working memory; attention allows certain stimuli be they sensory or cognitive to be given preference over other competing ones. Working memory refers to the keeping a limited amount of information usually for a few seconds to allow manipulation or use of that information for another task. Attention may be usefully categorized as follows.

- Focused attention—respond discretely to specific visual, auditory tactile stimuli.
- Sustained attention—maintain consistent behavioral response during continuous and repetitive activity.
- Selective attention—maintain behavioral or cognitive set in context of distracting or competing stimuli.
- Alternating attention—mental flexibility that allows individuals to shift their focus of attention and move between tasks.
- Divided attention—highest level of attention with ability to respond simultaneously to multiple tasks [78, 79].

*Monitoring*. This includes perseveration, and impersistence.

*Language*. This includes,Broca's aphasia (LH), expressive aprosodia (RH), transcortical motor aphasia, aphemia, and central aphasia.

*Emotional Control*. Clinical lesions studies have implicated in particular the orbitofrontal cortex as part of the neural network for emotional responses [80]. Patients with orbitoprefrontal and medial frontal regions were significantly impaired in both cognitive and affective empathy as compared to parietal patients and healthy controls and those with damage restricted to the prefrontal cortex, no matter which side, resulted in impaired empathy and lesions involving the right parietal lesions [81]. Subsequently from a registry analysis a much more widely distributed lesion site network impairs EI, in keeping with the extensive contemporarily appreciated neurobiological emotional network proposed by Pessoa (Table 6) [82]. Many different brain lesions may affect EI, including frontal, temporal, subcortical, and even subtentorial stroke syndromes with the strongest relationship (EI scores) pertained to the frontal and temporal regions [83] (Table 2). 

These are presented under headings and categories that we currently and traditionally see them. However, they are all frontal network syndromes that just happen to be treated by differing brain-related clinicians for historical reasons. Frontal network syndrome phenotypes may comprise of a mixture of the primary and secondary syndromes in various combinations. Clinical syndromes are also treated by different disciplines, with considerable overlap by psychiatry, neurology, neuropsychology, speech and language and physical medicine, and rehabilitation.

It may be readily appreciated that the construction of many clinical scales we use such as the FAB scale, the new consensus criteria for the behavioral variant of fronto-temporal lobe degeneration, and frontal network testing format of the coconuts are all derivatives of these core and secondary syndromes.

### 4.1. Hodological Perspectives, Hyperfunction and Improved Behavior

There are other frontal network presentations that are not readily classifiable under the above system. As a brain lesion may cause both hypo- or hyperfunction of a circuit or a lesion may cause hypo- or hyperfunction remotely (diaschisis) because of the hodological nature of brain function or connectomics. One theory termed the paradoxical functional facilitation proposes that one brain area reverses inhibition in other areas or results in compensatory augmentation, resulting in counter intuitive paradoxical improvement in certain functions function. According to this theory, increased originality requires inhibition of the left hemisphere and an intact right hemisphere [84]. Examples of such syndromes include the following.

(i) Emergent artistic ability in the setting of neurodegenerative disease has been reported in association with frontotemporal lobe disorders, stroke, Alzheimer's disease, Parkinson's, epilepsy, and migraine [85].
(ii) Delusional misidentification syndromes, seen particularly with right frontal stroke [86].
(iii) Increased humor, particularly after right frontal lesion such as stroke [87].
(iv) Loss of visual imagery in dreaming [88].
(v) Savant syndromes that may include the sudden, acquired prodigious, sudden, splinter, or talented subtypes [89].

## 5. Clinical: Diagnostic Tests—Beside and Metric

The time pressured nature of clinical practice, limited interview time, emergency room evaluation, and patient cooperation all place constraints on the nature of tests and how much testing can be performed. Hence it is useful to consider available tests in a time-orientated hierarchical manner. The overall decision on how to deploy more time consuming neuropsychological tests is detailed in the recommendations by the AAN neuropsychological testing guidelines [90]. An emerging viewpoint is that subcortical processes, mostly cerebrovascular, may be the most frequent cause of cognitive disorders, particularly in the mild cognitive impairment domain. The cognitive component in question is frontal network systems particularly executive function, attention, and working memory [91, 92].

### 5.1. Rapid (Bedside) Diagnosis

1. Montreal Cognitive Assessment (MOCA)—administration time. approximately 10–15 min [93].
2. Frontal Assessment Battery (FAB)—administration time approximately 15–20 min [94].
3. Executive Interview bedside test (EXIT)—administration time approximately 15–20 min [95].
4. Comprehensive cognitive neurological test in stroke (coconuts). Frontal Network System component—administration time approximately 20–30 min [96].
5. Metacognitive test—administration time approximately 20–30 min [97].

### 5.2. Computerized Screening Tests

  MindStreams [98]
  CANTAB [99]
  Cognistat [100]
  CNS Vital Signs [101].

### 5.3. Metric Tests

*Global Tests*

1. Delis-Kaplan Executive Function System (DKEF) [102].
2. Wechsler Adult Intelligence Scale (WAIS-IV) components [103].

*Clinical Syndrome Orientated, Questionnaire Based *

(3) Frontal Systems Behavior Scale (FRSBE) [104].
(4) Behavior Rating Inventory of Executive Function (BRIEF) [105].
(5) Frontal Behavioral Inventory (FBI) [106].

*Working Memory/Executive Function Tests*

(6) Trail Making Tests (comprehensive, trails A and B, color trails) [107].
(7) Letter and category fluency list generation [108].
(8) Wisconsin Card Sorting Test [109].
(9) Tower of London Test [110].
(10) Working memory tests (verbal and nonverbal).

*Emotional Intelligence Tests*

(11) Emotional Intelligence Quotient (EQ)—Bar-On et al. [111].
(12) Mayer-Salovey-Caruso Emotional Intelligence Test (MSCEIT) [112].

*Tests of Disinhibition/Inhibition*

(13) Stroop Neuropsychological Screening Test [113].
(14) Iowa Gambling Test [114].

*Autism *

(15) Autism Diagnostic Interview - Revised [115].
(16) Autism Spectrum Quotient [116].

*ADHD*

(17) Brown Attention Deficit Disorder Scales [117].

*Depression*

(18) CES-D (Center for Epidemiological Studies Depression Scale) [118].
(19) Beck Depression Scale [119].
(20) Hamilton Depression Inventory [120].

*Behavioral Neurological Tests*

(21) Faux Pas Test [121].
(22) Reading the Mind in the Eyes Test [122].
(23) Hotel Task [123].
(24) Multiple Errands Test [124].
(25) Ambiguous Figures Test [125].

*Creativity Tests*

(26) Torrance Test of Creative Thinking [126].

*Other Tests That Predominantly Assess Frontal Network Systems*

(27) Visual Search and Attention Test [127].
(28) Rey Complex Figure Test [128].

*Clinical Tests—Qualitative without Normative Data*

(29) The Executive Control Battery [129].

*A Novel Approach*

(30) The metacognitive battery incorporating neurological, neuropsychological, and neuropsychiatric syndromes [97].

*Aphasia Tests Useful in Motor Aphasia, Dysnomia, and Aphasias in General *

(31) Western Aphasia Battery [130].
(32) Boston Diagnostic Aphasia Evaluation [131].

*Elementary Neurological*

  Olfaction (The Smell Identification Test, Sensonics [132]
  Gait
  Incontinence
  Primitive reflexes (grasp reflex, palmomental reflex, sucking reflex)
  Volitional eye movements.

## 6. Neuropathological States

Due to the expansive frontal subcortical circuits and their open connections, it may be readily appreciated that the frontal lobes connect with all other regions of the brain including the cerebellum and brainstem [133]. Clinical lesion studies have repeatedly shown that no matter where the brain lesion is, whether subcortical gray matter, subcortical white matter, cerebellum, brainstem, or even parietal and occipital lobe, a greater or lesser degree of frontal systems syndrome is present [1–7, 134]. Even transient ischemia has been associated with a transient frontal network syndrome [135].

An appreciation of the tropism of the various neuropathological states is important. FTD is relatively confined to the frontal and anterior temporal lobes, similarly herpes simplex encephalitis. However, cognitive vascular disorders (CVD), disorders of white matter such as the leukodystrophies and CADASIL, vasculitis TBI, MS, most of the toxic metabolic encephalopathic all tend to affect the frontal subcortical networks in a more diffuse fashion and hence present with inattention, dysexecutive syndrome, and dysmemory as the hallmark signature syndromes, considered the most common presentations of frontal network syndromes [133]. Many of these will have varying degrees of neuropsychiatric syndrome admixtures most commonly depression and anxiety and perhaps less often disinhibitory behavior, irritability/aggression, obsessive-compulsive disorders, and adult onset ADHD.

**Figure 9:**
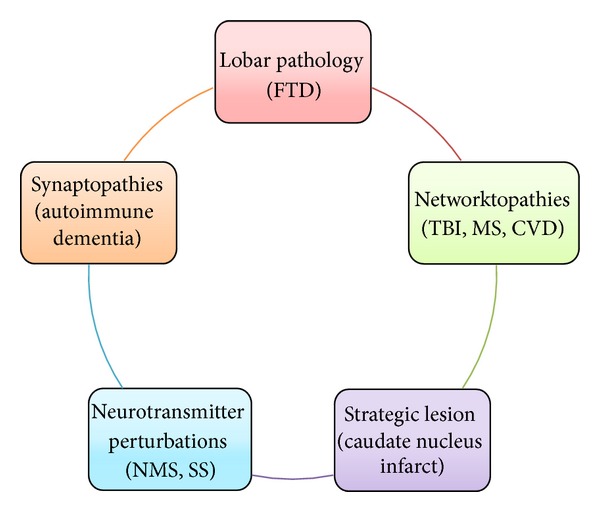
Frontal network syndromes may be caused by focal or diffuse processes with differing pathophysiologies.

Syndromes affecting the modulatory systems of the brain may also present with cognitive alterations. The serotonin toxidrome may present with barely perceptible symptoms to coma with cognitive, somatic, and autonomic manifestations [136]. The cognitive features may include hypomania, agitation, hallucinations, the autonomic hyperthermia, hypertension, tachycardia, diarrhea, and somatic features: myoclonus, tremor, and hyperreflexia [137]. Pathophysiologically, there is an increase in cerebral serotonin or overstimulation of the 5HT 2A receptors, often due to MAOI's in combination with SSRI, SNRI, TCA, appetite suppressants, or opioids [138]. The diagnosis remains a clinical one. Similarly with the neuroleptic malignant syndrome, cognitive changes, autonomic instability, tremors, muscle cramps, tremors, and elevated creatinine phosphokinase are noted. The fever is caused by a hypothalamic dopamine receptor blockade and the muscular effects due to blockade of the D2 receptor and the pathophysiology due to low dopamine or dopamine receptor blockade with sympathoadrenal hyperactivity [139]. With the malignant hyperpyrexia syndrome, exposure to anesthetic drugs such as succinylcholine, a neuromuscular blocking agent, halothane, or desflurane, an abnormal muscular activity is induced with hyperpyrexia and circulatory collapse that can be fatal. The inheritance is autosomal dominant, usually for the ryanodine receptor (gene RYR1) located on the sarcoplasmic reticulum and opens in response to an increase in intracellular calcium which is exaggerated in this condition. There is also a relationship with central core myopathy [140]. The cholinergic toxidrome may take the form of anticholinergic or cholinergic toxidrome. Both have a similar presentation including cognitive autonomic and muscular symptoms and signs but with some important differences such as tachycardia, mydriasis urinary retention, hyperthermia with the former and diarrhea, urination, hypothermia miosis, and bradycardia with the latter. Both also present with psychosis, seizures, hallucinations, delirium, and myoclonus [141]. PAIDS (paroxysmal autonomic instability and dystonia syndrome) is usually seen after significant traumatic brain injury and also presents with a combination of cognitive alterations, autonomic abnormalities, and, in this instance dystonias rather than muscular rigidity and tremor [142] (Figure 9).

**Figure 10:**
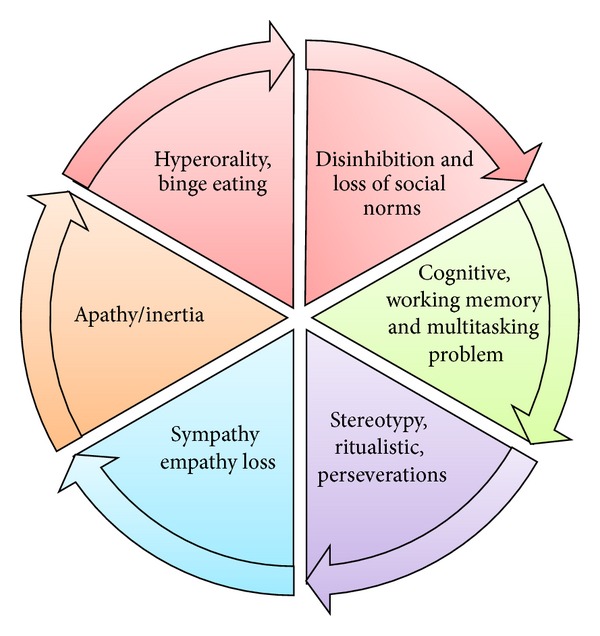
FTD phenotypes. New International criteria for diagnosis of FTD.

Frontal syndromes or frontal network syndromes may come to attention by way of the patients symptoms, a clinical syndrome elicited clinically or primarily by neuroimaging findings bearing in mind that this part of the brain has sometimes been termed clinically silent (Tables 3–5 and Figure 10). 

### 6.1. Frontotemporal Lobe Degeneration

Frontotemporal lobe degeneration as a generic term rather than dementia is recommended because many remain in a category of MCI for a long time before frank dementia supervenes. Because of the protean manifestations and long duration of decline, sometimes for decades, diagnostic difficulty is the rule. Eventually neuroimaging may reveal focal degeneration of frontal insular and temporal lobes although the basal ganglia and spinal motor neurons may also be involved and the presentations may accordingly be initially one of the parkinsonian dementia syndromes (CBD, PSP, and even DSDB) and MND. Hence it is useful to consider FTLD in the context of other primary dementias [143] (Table 6).

### 6.2. Epidemiology

FTD is more common than AD in those <60 years and the prevalence is about the same as AD in 60–70 group and there are reports of FTD presenting in the third decade. The most common subtype is bv-FTD and a rapidly progressive FTD is seen in association with motor neuron disease with a mean survival of approximately two years. By comparison, the survival of bv-FTD is between 6–8 years with the language variant subtypes currently demonstrating the longest survival times of 8–12 years [143–145].

### 6.3. Clinical Presentations

People with FTLD have a particularly protean clinical presentation from subclinical to requiring institutionalization. The symptoms and signs initially are subtle, often undiagnosed for years and frequently misdiagnosed as bipolar disease, mania, obsessive compulsive disorder, personality disorder, and depression. Dysexecutive function is not specific to FTD and is also seen with other dementias including AD, whatever the emotional impairment is. Memory complaints are unusual. When the pathology is left sided, language impairment is a major clue and all present with word finding problems. However, in logopenic progressive aphasia (LPA), the person retains the so-called “islands of normal speech” without dysarthria [145].

### 6.4. Clinical Diagnostic Criteria

**Figure 11:**
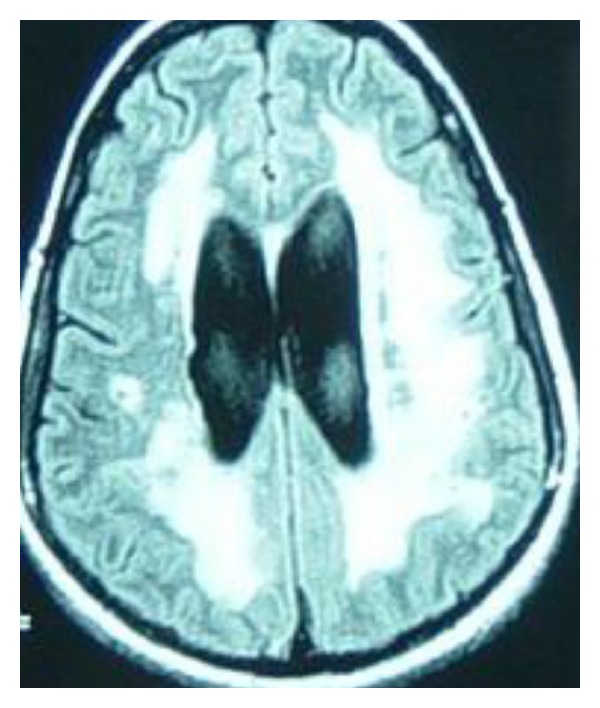
Leukoaraiosis: A common causes of frontal network syndromes.

The criteria of Neary et al. [146] have been augmented by the revised international consensus criteria of Rascovsky et al. which are presented in abbreviated form (appendix 2) in [147]. In brief, the major features include (Figure 11) the following

(i) progressive deterioration of behavior and/or cognition by observation,
(ii) early behavioral disinhibition,
(iii) early apathy,
(iv) early loss of sympathy or empathy,
(v) early perseverative, stereotyped, or compulsive/ritualistic behavior,
(vi) hyperorality and dietary changes,
(vii) executive deficits with relative sparing of memory and visuospatial functions.

### 6.5. Pathology of FTD

TAR DNA binding protein 43 (TDP-43) was identified as the major ubiquitinated protein, with positive inclusions in neurons and glia, occurring in FTD and approximately half of FTD patients have TDP 43 inclusions due to mutations in the progranulin gene found in the frontal and temporal neurons and dentate gyrus. Most the remainder is due to tau inclusions caused by mutations in the tau gene. Other pathological-clinical associations of TDP 43 include the FTD—MND association, SV is mostly linked to TDP 43, and hippocampal sclerosis (frequently seen in FTD) and is usually associated with TDP 43. The bv-FTD may be associated with tau or TDP 43. PNFA is more likely due to tau pathology. About one quarter of progressive Lewy body disease patients may also show TDP 43 inclusions [148–152].

Overall, in FTD the pathology is, therefore, either a tauopathy or TDP 43 proteinopathy with a small number associated with other pathologies (Table 7). Tau is a microtubule-associated protein that is integral to microtubule assembly and in stabilizing microtubules [153–155].

Histologically, von Economo neurons, a relatively unique spindle-shaped cell found in mammals of such as cetaceans, hominoids, and elephants, are found in the anterior cingulate cortex and fronto-insular regions and are thought to be important in social networks. These cells selectively degenerate in the early stages of bv-FTD. Neurochemically, the presentations of FTD may be due to a significant reduction of serotonin receptors in the cingulate, orbitofrontal, and insular regions [156].

### 6.6. Genetics

Overall, 10% of FTD cases are associated with an autosomal dominant pattern of inheritance with the rest sporadic. Both tau and progranulin mutations have been linked to chromosome 17, and other genetic loci linked to FTD occur on chromosome 3 and 9. Both tau and progranulin mutations cause inclusions in neurons and glia. In general, bv-FTD has the strongest inheritance, SV the less, and PNFA intermediate [157].

#### 6.6.1. Clinical Subtypes

(1) Behavioral variant.
(2) Progressive non fluent aphasia (left perisylvian degeneration associated with tau pathology).
(3) Semantic variant (anterior temporal degeneration associated with TDP 43 proteinopathy but about 10% have AD).
(4) Logopenic progressive aphasia (angular gyrus of parietal lobe usually associated with AD pathology).

#### 6.6.2. Associated Syndromes

Other neurological deficits may be elicited including peripheral neuropathy, parkinsonism, apraxia, and gaze abnormalities which would support the existence of one of the following syndromes.

1. FTD and Parkinsonism and corticobasal-ganglionic syndrome.
2. FTD and ALS—more common with bulbar onset ALS than limb onset ALS.
3. FTD and PSP—present vertical gaze impairment, with early falls axial rigidity.
4. CBD: asymmetric movement disorder with alien hand syndrome and cortical sensory loss. Rather than just a primary disorder, CBD may be caused by secondary conditions such as FTD, AD, and Creutzfeldt-Jakob disease. For example, CBD, PSP, and half of people with FTD have MAPT (microtubule associated protein tau) [155].

### 6.7. Investigations

#### 6.7.1. Clinical Cognitive/Behavioral

It is important to employ specific frontal behavioral batteries such as FBI and FRSB as neuropsychological cognitive testing may be normal with a distinct discrepancy in the relative absence of amnesia and visuospatial impairment.

#### 6.7.2. Neuroimaging

Structural (MRI/CT) may reveal frontal or temporal atrophy and functional (PET, SPECT) revealing frontotemporal abnormality much earlier with frontal and/or anterior temporal lobe hypometabolism or hypoperfusion, respectively. A study using MR perfusion scanning revealed similar results and may prove to be more practical [158].

#### 6.7.3. Laboratory

CSF tau and ratio of tau to A-beta 42 are significantly lower in FTD than in AD with a sensitivity of 79%–90% and specificity of 65% to 97% [159]. 

#### 6.7.4. Treatment

In brief, pharmacological management may be employed using serotonergic agents for decreasing obsessions disinhibition, overeating, and repetitive behaviors, as exemplified by the randomized control trial of Trazodone. Atypical antipsychotics for agitated behavior and avoidance of cholinesterase inhibitors is also recommended as the latter may cause agitation [160]. Investigational treatments include both tau- and PGRN- based approaches. For example, inhibition of tau kinases to prevent tau hyperphosphorylation may be accomplished using lithium chloride or valproate. Prevention of polyubiquitination to decrease tangle maturation by using HSP-90 and so antagonizing tau fibrillation or stabilizing microtubules using paclitaxel or anti-inflammatory agents is another possible avenue. Low progranulin levels might be treated with replacing progranulin [161].

### 6.8. Cerebrovascular/Cognitive Vascular Disorders: An Example of a Networktopathy with One or More FSC Affected

The spectrum of cognitive vascular disorders cerebrovascular disorders includes the following.

*Cognitive Impairment—No Stroke*

  Brain at risk stage (risk factors only: hypertension, diabetes, dyslipidemia)
  Transient ischemic attacks and cerebral infarct with transient symptoms.

*Cognitive Impairment—Subcortical Infarct*

**Figure 12:**
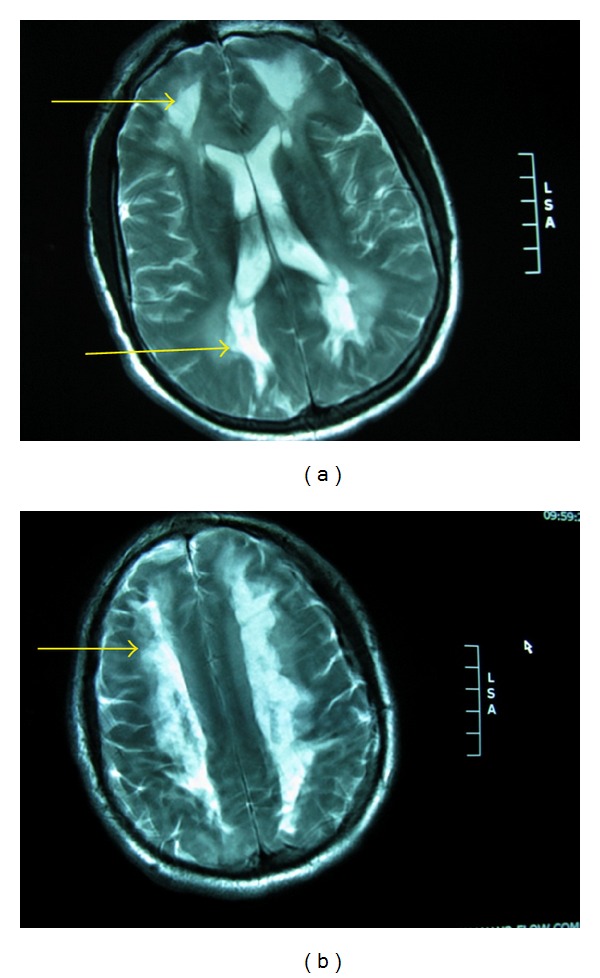
Watershed infarction (arrows).

**Figure 13:**
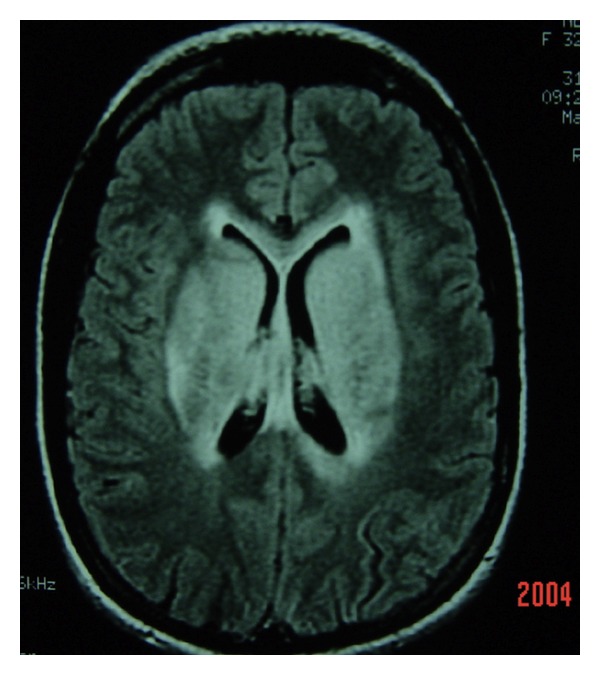
Tegmentothalamic infarction due to deep venous system thrombosis postpartum.

 Strategic infarct: caudate, thalamus, basal ganglia
 Leukoaraiosis (Figure 11)
 Watershed infarction (Figure 12)
 Deep venous system tegmentothalamic lesions (Figure 13).

*Cognitive Impairment—Cortical Infarct*

**Figure 14:**
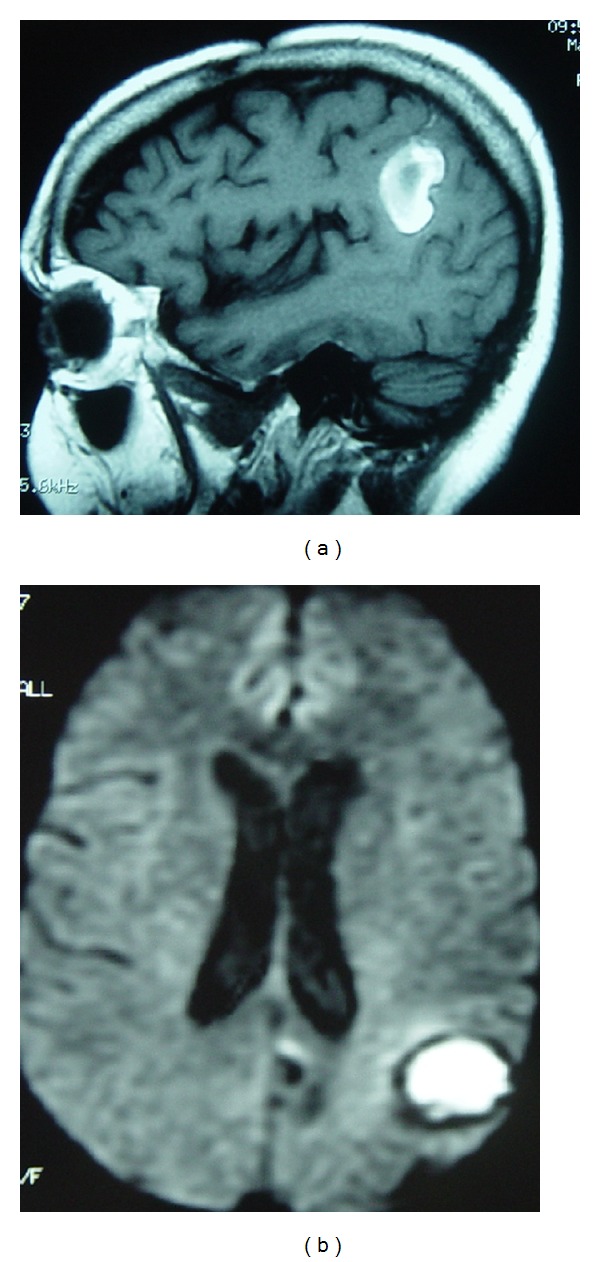
Discreet hemorrhage in left angular gyrus region presenting with Gerstmann's syndrome.

 Left angular gyrus (Figure 14), right temporal lobe, frontal.

*Cognitive Impairment—Subtentorial Stroke*

 Brainstem and cerebellum.

*Multiple Infarcts*

 
*Vascular Dementia.* Most of the various cerebrovascular syndromes such as small vessel disease, leukoaraiosis, and vasculitis as well as multiple sclerosis and traumatic brain injury affect the brain more diffusely. Consequently the prototypical deficits involve attention, memory, and executive functioning. This is important to consider as many of these patients have a considerably reduced attention span (and some may be irritable or irascible at the same time due to their disease process), and this greatly impacts the mode of testing. Extensive neuropsychological testing in such patients is not usually practical and one of the shorter batteries may suffice. However, decline in memory is strongly associated with AD and decline in executive function is strongly associated with CVD [162].

The origin of cognitive impairment in the context of vascular disease and whether neurodegenerative disease might be implicated; the following are considered.

- The pattern and severity of cognitive impairments.
- The size and location of infarcts including symptomatic infarcts, silent infarcts, and leukoaraiosis.
- The severity of atrophy patterns in particular hippocampal atrophy and pattern of atrophy of frontal versus parietal.
- Do the vascular lesions adequately explain the cognitive impairment?
- If the vascular lesions do not adequately explain the cognitive impairment, then neurodegenerative disease is likely present as well.

In studies of neuropsychological series of vascular cognitive impairment and AD, the former had executive dysfunction and less impairment in verbal episodic memory. There were no differences in language, constructional abilities, and attention [163, 164].

The current definition of dementia is memory centered, requires ADL impairment, and does not emphasize the predominant executive dysfunction of the CVD subtypes. For minor cognitive deficits due to vascular disease, the term VCI-ND has been recommended (cognitive deficits that do not meet criteria for dementia but impair minor chores) [165].

Neuroimaging has been helpful in differentiating AD and CVD. More WM changes or leukoaraiosis and less medial temporal lobe atrophy are seen in CVD when compared to AD although overlap occurs. Two types may be discerned; when the WM lesions are distinct and separated from ventricles, this tends to be more specific for vascular dementia. When the WM hyperintensity is periventricular, this tends to support a neurodegenerative rather than vascular process [166]. A formidable number of conditions can present with white matter hypertensities on MRI brain scan (Table 8). 

Leukoaraiosis may affect one, some, or all of the FSC and quantification will likely become more important in the future; apart from neuroimaging software capabilities, the Junque classification system (Table 9) is a very useful manner of quantifying and measuring over time. 

In general the leukoaraiosis (LA) on MRI can be interpreted as follows.

(1) Mild MRI LA is correlated with markedly lower scores on episodic memory compared to working memory and is a neuropsychological feature associated with AD.
(2) Moderate MRI LA correlates with both amnesia and executive dysfunction.
(3) Severe MRI LA correlates with significantly lower scores on working memory and executive dysfunction [167].

These findings have treatment implications with the milder forms more amenable to cholinergic therapy and the more severe forms might perform better with dopaminergic therapy. 

#### 6.8.1. Neuropathology of Vascular Lesions

- Cerebral infarcts
- Lacunes
- Microinfarcts—up to 5 mm
- Widening of perivascular spaces
- Incomplete infarction
- Leukoencephalopathy—associated with SVD.
- Laminar necrosis selective involvement in the third and fifth layers.
- Granular atrophy patches of gray matter between 2 or 3 arterial territories
- Lobar hemorrhages—linked to A-ß angiopathy and AD
- Small hemorrhages—association HTN at corticosubcortical junction (slit hemorrhages) and BG. The latter are called type II lacunes [168].

#### 6.8.2. Neurobiology of Cognitive Vascular Disorder

Leukoaraiosis (LA) may interrupt the neurotransmitter modulatory systems such as aminergic or corticostriatal and thalamocortical networks. Patients with moderate MRI LA may respond better to Aricept especially with Junque LA scores of ≥10 compared to those with minimal MRI LA. There may be at least moderate MRI LA in context of a dysexecutive syndrome, which may be a marker for the relative preservation of cholinergic neurons. Delayed recognition memory measure is relatively preserved in subcortical vascular disease compared to AD [69]. Perhaps what is important in this context is that the cerebral vasculature may be observed in real time by fundoscopy. The following were predictive of lacunar stroke: narrower central retinal arterial artery equivalent, wider central retinal vein equivalent, focal arteriolar narrowing, and arteriovenous nicking [169].

Overall, a mixed dementia is the most common type; one that has been aptly termed a vascular tsunami (Hakim). Population-based autopsy studies indicate that less than 50% of patients have pure AD and many people diagnosed with AD may in fact have VCD. The neuropathology of AD and VCI may coexist and influence each other and 8 of 10 of the traditional vascular risk factors also pertain to AD such as hypertension, hyperlipidemia, hyperhomocysteinemia, APOE2, and APOE 4 [170]. Importantly, treatment of vascular risk factors is associated with slower decline in AD with no CVD [167, 171].

### 6.9. Frontal Variant of Alzheimer Disease (AD)

AD is normally regarded as disease process afflicting the posterior brain, namely, parietotemporal regions with a clinical correlate of dysmemory, visuospatial impairment, geographical disorientation, and only much later behavioral abnormalities that are characteristic of FNS. The usual AD variants include a primary progressive aphasia and visuospatial and posterior cortical atrophy syndrome subtypes or variants. Reports indicate that about 14%–17% of AD patients have nonmemory presentations as an atypical subtype [172]. In addition, executive function is not considered as a component of AD at least in mild-to-moderate disease [173]. However, a subpopulation of AD patients may present with an FNS early on including all three principal frontal syndromes of abulia, dysexecutive, and disinhibition. The neurobiology may be a disturbance of the frontal subcortical systems but the underlying etiology remains to be determined. One study found a 10-fold greater neurofibrillary tangle pathology in BA 8 of the frontal lobes [174]. Other explanations may include white matter disease, coexistent Lewy body disease, and other cerebrovascular pathologies that influence the clinical presentation of the AD as a frontal variant [175, 176].

### 6.10. Multiple Sclerosis (MS)

As a pathophysiological process with a few or numerous subcortical plaques, MS is a process that may impair one or more of the FSC's. Recent studies cite a data indicating that 43%–72% of patients with MS are considered to have cognitive impairment [177]. The correlation with MS plaques as measured by standard MRI is not always present. MS plaques frequently lie adjacent to the ventricles and so tend to interrupt the long association fibers in the cerebrum such as the fronto-occipital fasciculus, the superior and inferior longitudinal fasciculi. 

A recent study underscoring the important neurobiology of the frontal subcortical tracts employed frontal neuropsychological as well as behavioral neurological metric tests in comparison to diffusion tensor imaging (DTI) of the frontal subcortical systems. Tests sensitive to the orbitofrontal and cingulate regions of the frontal lobes were used including the Hotel Task Test, Iowa Gambling Test, Faux Pas Test, Multiple Errands test, and Reading the Mind in the Eyes Test. DTI evaluations were performed in the frontomedial, frontolateral, orbitofrontal anterior cingulate and a significant difference was found with lower FA values in the FM and FL in the MS patients compared to controls. A significant correlation was also found with loss of fiber integrity in the frontolateral regions and an impairment on the Hotel Task test and Multiple Errands test [178].

When cognitive impairment in MS is evaluated, the symptoms elicited are principally inattention, abulia (apathy), dysexecutive syndrome, dysmemory (working memory), and disinhibition (inappropriate jocularity). Elicitation of these symptoms is important in that each of these is potentially treatable. Stimulant therapy with dopaminergics that pertain to the first four and sodium valproate or carbamazepine is often effective for treating disinhibition syndromes.

### 6.11. Traumatic Brain Injury (TBI) and Concussion

TBI is associated with a complex syndrome characterized by cognitive (memory, attention and executive problems), elementary neurological symptoms (headache dizziness, vertigo, imbalance), and neuropsychiatric impairment (anxiety, depression, irritability, irascibility, mania, disinhibition, impulsivity). Symptoms may be relatively mild to severe and may be present with normal neuroimaging and even anatomical pathology leading to frequent misdiagnosis and under appreciation of the severity of the syndrome. Key to understanding the complexity of these syndromes is the current understanding of the pathophysiology (Table 10). 

The dramatic advances in neuroimaging and alterations in the biochemistry and vascular system might best be described as a networktopathy with neurotransmitter and vascular perturbations that often escape anatomical imaging. The realization that vasospasm is a frequent accompaniment during concussion and TBI has led to the transcranial Doppler study initiatives as a novel way of noninvasive monitoring [179]. Default mode network imaging may prove to be the most sensitive diagnostic tool yet in diagnosis [180].

### 6.12. Alcohol Excess

The frontal lobes are particularly susceptible to the effects of alcohol, as revealed by recent magnetic resonance spectroscopy studies [181]. The hippocampal CA1 and CA3 regions are also affected, particularly in animal models [182]. In addition, all of the following alcohol related cerebral conditions can affect cognitive functioning in particular frontal systems of working memory, attention, and executive function.

- Alcohol intoxication—coma, pathologic intoxication, alcoholic blackouts/memory loss.
- Abstinence or withdrawal syndromes.
- Nutritional disease (Wernicke Korsakoff).
- Cerebrovascular infarcts and hemorrhages.
- Cardiomyopathy.
- Cerebellar degeneration.
- Marchiafava-Bignami disease.
- Central pontine myelinolysis.
- Alcoholic dementia.
- Cerebral atrophy.
- Neurologic conditions secondary to liver cirrhosis, portal shunts.
- Traumatic brain lesions during intoxication.

### 6.13. Normal Pressure Hydrocephalus

Previously characterized as a subcortical dementia, this syndrome is still diagnosed by the clinical triad of cognitive, gait impairment and urinary incontinence. The predominant cognitive presentation is a frontal syndrome characterized by dysmemory, dysexecutive syndrome, inattention, and speed of information processing slowing. The frontal lobe syndrome or FNS is much more profound than that seen with AD and the memory disturbance is relatively mild with NPH, being much more severe with AD. The pathophysiology includes damage to FSC, corpus callosum, thalamus, basal ganglia, and hippocampus. The cognitive impairments respond to appropriate shunt procedures but are usually less so than the improvement in gait impairment. [183]. Recently the importance of specific assessment of executive function assessment has been proposed as this can be differentially improved by shunting relative to other cognitive impairments [184]. Neuroimaging with PET (or SPECT which measures perfusion rather than metabolism) has been shown to be the best diagnostic tool and a frontal hypometabolism (in NPH) as opposed to a posterior parieto-occipital hypometabolism (in AD) has been shown to differentiate NPH from AD better than cisternography [185].

### 6.14. Autoimmune Disorders and Limbic Encephalitis as Examples of Synaptopathies

A reappraisal of autoimmune conditions associated with cognitive impairment and at times frank dementia has led to the concept of immunotherapy responsive dementias and encephalopathies. In addition to being treatable and reversible causes of dementia, they may account for up to 20% of the so-called young dementia patients (<45 years) [19]. The cognitive profile includes the usual combination of FSC impairments in speed of information processing, dysmemory, and behavioral abnormalities typically of a fluctuating nature as well as agitation, hallucinations, and seizures [186].

The finding that 1 alpha dendrotoxin antibodies against VGKC noted in patients with Morvan's disease, limbic encephalitis, and neuromyotonia completely changed the understanding from limbic encephalitis being a rare paraneoplastic condition with poor outcome and associated with anti-Hu, anti-Ma2- or CV2/CRMPS antibodies. Autoimmune dementia syndromes (AID) in general are disorders with antibodies against synaptic proteins such as NMDA, AMPA, and GABA-B receptors and are treatable (Table 11). The identification of a number of neural specific autoantibodies such as voltage gated potassium channel (VGKC) antibodies has increased the number of phenotypic presentations of AID. The diagnosis is made by establishing cognitive impairment or an encephalopathic state with clinical, radiologic, or serologic evidence of autoimmune etiology. Other causes of dementia require exclusion and a beneficial response to an immunotherapy trial [30, 187]. 

In addition to the specific antibody tests, cerebrospinal fluid analysis is useful with the following parameters regarded as support for AID, raised protein especially over 100 mg/dL, pleocytosis, oligoclonal bands, and IgG index elevation. Cancer screening is important with computerized chest, abdomen, and pelvis required in all patients, mammography in women and prostate specific antigen and testicular ultrasound in men. Treatment options for AID include intravenous methylprednisolone (1000 mg × 3–5 days then weekly for 6–8 weeks), IVIG if seropositive for GAD 65 or IA2 autoantibodies with plasma exchange cannot tolerate steroids or IVIG. Long-term therapy is indicated in responders that may require steroid sparing inhibitor such as azathioprine, mycophenolate, cyclophosphamide, or methotrexate [29, 187].

### 6.15. Cerebral Vasculitides, Infectious Disorders, Autoimmune Disorders, and Chronic Inflammatory Disorders

These conditions share several features in that they involve the brain diffusely often with small or microscopic lesions, have relatively covert onset and often subtle signs and syndromes, and may often be overlooked as consequence. The FSC's are affected as well as the open-ended connections to the posterior parts of the brain, the brainstem, and cerebellum. In addition to standard anatomical brain imaging, CSF analysis is required and very often, functional imaging with SPECT or PET brain scanning, at times magnetic resonance spectroscopy and less often brain biopsy. The challenge is usually in considering or entertaining these disorders in the first place as antibody testing, CSF analysis, cerebral catheter angiography and at times brain biopsy as indicated are often the diagnostic.

Many pathological processes may have as their earliest manifestations some degree of cognitive impairment, most commonly involving the frontal network systems with systemic and meningeal irritation manifestations either absent or appearing much later in the illness. Hence frontal network syndromes may be the first sign of a potentially treatable disorder, which if missed, is devastating. With CSF analysis of dementia, currently a nonroutine investigation, it is possible to ascribe a slowly dementing state to the most common disorders such as AD or FTD and miss a treatable condition such as chronic cryptococcal meningitis. This has indeed been reported several times and may present a tip of the ice berg phenomenon [188–190]. Others seen much less frequently include *Candida, Aspergillus, Blastomyces, Coccidioides*, and *Histoplasma* (Table 12).

### 6.16. Vasculitides

In the largest series to date (*n* = 101) of cerebral vasculitis, cognitive impairment in general was present in over three quarters of the patients with altered cognition in 50% and aphasia in 28% of patients [191]. As with other subcortical and more diffuse brain processes, inattention, dysexecutive function, and working memory problems are the most common cognitive disturbances. Laboratory (ESR, CRP), cerebral angiography (often four vessel catheter cerebral angiography) and at times brain biopsy are required for diagnosis but the most important clinical error is not to entertain the diagnosis in the first place in the appropriate clinical context (Table 13).

*Conditions That May Present with Focal or Diffuse Arterial Narrowing and in the Differential of Vasculitis*

 Radiotherapy
 Vasospasm: acute hypertension, migraine, benign reversible cerebral angiopathy, or posterior reversible encephalopathy syndrome (PRES)
 Lymphoma of the central nervous system
 Intracranial dissection: traumatic, spontaneous, and fibromuscular dysplasia
 Intracranial atherosclerosis
 Recanalizing embolus
 Moyamoya disease and syndrome
 Tumor encasement due to pituitary adenoma or meningioma
 Sickle cell anemia
 Neurofibromatosis [192, 193].

## 7. Neuroradiology

### 7.1. Anatomical and Functional Imaging

In the context of FSC syndromes, the first step is to exclude emergency neurological conditions such as cerebral infarct, HSV-1, meningitis, subdural hematoma, and mass lesions using anatomical imaging with CT and multimodality MR imaging. Thereafter the pursuit of underlying etiological processes is attended often requiring and complemented by functional imaging usually such as DTI, f-MRI, ^18^Fluorodeoxyglucose position emission tomography (^18^FDG-PET) brain, single-photon emission computed tomography (SPECT), brain PET Pittsburgh compound B (PIB), and PET receptor (Dopa) imaging (Tables 14 and 15). ^18^FDG- PET brain in particular has been an important tool in the early diagnosis of mild cognitive impairment (MCI) and in differentiating types of dementia, with frontotemporal disorders (FTD) and Alzheimer's disease (AD) [194]. Functional imaging is increasingly able to detect pathology, long before the clinical state emerges with PET brain imaging being the most accurate diagnostic method for most common dementia categories [195]. PET brain scan patterns reliably differentiate the major dementia subtypes including the AD variant, posterior cortical atrophy syndrome (Benson syndrome) [196], FTD (frontotemporal hypometabolism), and AD (temporal, parietal, posterior cingulate hypometabolism) being relatively easily identified. There are also overlap syndromes such as AD and cognitive vascular disorders (CVD), the frontal variant of Alzheimer's disease, and bv-FTD which cannot be differentiated easily clinically [197]. Other conditions that present predominantly as an FNS syndrome include autoimmune dementias, toxic dementias, HIV dementia, and the prefrontal atrophy secondary to chronic stimulation of the pain matrix (chronic pain syndrome) [198–201]. Positron emission tomography (PET) [202–204] and functional magnetic resonance imaging (fMRI) [205, 206] implicate the anterior cingulate cortex (ACC) and posterior cingulate cortex (PCC) having key roles in processing of pain perception [207].

MR perfusion scanning gives similar information to PET brain scanning, and, being based on MRI techniques, lack of radiation may give this modality preference in the near future [208]. 

### 7.2. Resting State Network (RSN) or Intrinsic Connectivity Networks (ICN) Imaging

The Default Mode Network (DMN), for example, reflects the basal or default mode activity of the brain (without activation procedures). Regions metabolically active include the posterior cingulate, the precuneus, lateral parietal, lateral temporal, and medial frontal areas. Hence the DMN is active during rest and becomes less active during task engagement. DMN connectivity disruption has been documented in AD, FTD, epilepsy, autism, schizophrenia and depression (Table 16) [34]. Interestingly, the distribution of the DMN impairment is similar to the fibrillar amyloid deposition seen with AD (amyloid PET scanning) [35]. DMN disruption was accurate in identifying major depression with a 94% correct classification with the amygdala, anterior cingulate cortex, parahippocampal gyrus, and hippocampus exhibiting the highest discriminative power in classifying major depression [34]. Using fc-MRI of the DMN and other RSN such the salience (for FTD), as well as attentional networks, allows RSN patterns to differentiate AD and FTD [35]. 

### 7.3. Neurotransmitter and Neurotransmitter Receptor PET

In AD, for example, cholinergic (nicotinic receptors) and dopaminergic systems measurements have revealed increased ^11^C nicotinic binding sites associated with cognitive improvement after rivastigmine for 3 months [209]. In Parkinson's disease using ^11^C methyl-4-piperidyl acetate (MP4A), dopaminergic system imaging with ^18^F fluorodopa (FDOPA) showed decreased uptake in the striatum [210]. 

### 7.4. Diffusion Tensor Imaging (DTI)

DTI has become the imaging modality of choice to objectively quantify the anatomical pathology which predominantly affects the fiber tracts that occur with traumatic brain injury and multiple sclerosis, for example, often with normal standard MRI scans [211, 212]. 

## 8. Treatment options

### 8.1. Overview

There are a number of pathomechanisms that are associated with brain injury and an understanding of these may lead to avenues of improved brain function after injury. In the endeavor of promoting improvement after brain injury, consideration needs to be given to the following:

1. augmenting and supporting mechanisms of spontaneous recovery,
2. avoiding interventions (particularly medications) that may worsen the condition,
3. pharmacotherapy—mainly the ascending monoaminergic systems,
4. behavioral therapies—using the top down influence of the prefrontal cortex,
5. overcoming inhibitory influences after injury (MVF-type therapy).

Pharmacotherapy is mainly concerned with the neuromodulatory systems, which are mainly concerned with adjusting signal-to-noise ratios and so influence processing [213]. Neuromodulation may be associated with augmenting, diminishing, or prolonging signaling in neuronal networks. There is also a top down regulatory control over the ascending modulatory systems from the PFC to the brainstem neuronal cells groups of NE, DA, 5HT, and Ach [214, 215].

Information gleaned mostly from animal models have revealed the cellular and molecular responses to brain injury. Currently known processes that are involved in spontaneous recovery in the stroke model, for example, include the following.

*Cellular*

  Increased angiogenesis
  Increased synaptogenesis
  Increased dendritic branching and spine density
  Increased neuronal sprouting.

*Receptor*

  GABA downregulation
  Increased N-methyl-D-aspartate receptor binding.

*Molecular*

  Increased growth factors
  Increased cell cycle proteins
  Increased growth associate proteins
  Increased inflammatory markers
  Hyperexcitability with long-term potentiation facilitation [216].

Future treatment strategies have been proposed for the stroke model but these may be equally applicable to other brain pathologies (Table 17).

#### 8.1.1. Basic Science Evidence from Animal and Human Studies of Treatment Effects on Core Frontal Functions

An important principal revealed by basic science animal models has been the realization that neurotransmitter systems function in phasic as well as tonic modes [217]. This applies to the modulatory ascending systems and correlate with the concept of a bell-shaped curve or the Yerkes-Dodson inverted U-shaped response seen in animals and humans. This psychological concept relates to the task performance with the horizontal axis representing level of arousal and vertical axis represents a persons particular performance with the peak or top of the bell being the site of optimal performance [218]. This implies that level of monoaminergic function optimal for one particular task may not be so for another task and may be either be sub- or supraoptimal [219]. For example, different levels of norepinephrine (NE) may affect different NE receptors, with moderate levels of NE release affecting high affinity alpha 2 A receptors, and even higher NE levels as encountered during stress, involve alpha 1 adrenoreceptors and beta adrenoreceptors [219]. These receptors have opposing functions in the PFC, the former improving and the latter impairing PFC function. The same applies to D1 and D2 receptors where different levels of presynaptic dopamine levels may either improve components of cognition or impair others [220, 221]. Both NE and DA are also considered to have complementary actions affecting cognition function in the PFC as has been reported with respect to spatial WM function, for example [222]. The specific mechanisms of the monoamines of regulation of working memory have implicated the hyperpolarization activated cyclic nucleotide gate cation channels (HCN channel) which are localized on heads and necks of dendritic spines near incoming synapses in the superficial layers of monkey PFC. These layers form the corticocortical networks [223]. The other functions of the monoamines on the PFC include excitatory and neuroplasticity effects [224, 225]. The NE component has been associated with sustained attention when in its phasic mode and distractibility in its tonic mode in nonhuman primates performing a go-no-go visual attentional task. In addition, single unit recordings of the locus coeruleus was associated with optimal performance on a go-no-go visual target detection paradigm and in rhesus monkeys was correlated with phasic firing of NE cells [226].

#### 8.1.2. The Contribution of Genetics to Potential Treatment of Neuropsychiatric States

These include alterations in gene encoding molecules associated with glutamate signaling, cortical development, and the ascending monoaminergic systems as follows.

 DISC 1 (disrupted in schizophrenia 1): major susceptibility gene for mental illness including schizophrenia, bipolar disease, and depression.
 DISC1 interacts with phosphodiesterase 4B (PDE4B) and impaired DISC 1 function likely leads to overactivation of cAMP-HCN signaling and weakening of the PFC network connections [223, 227].
 RGS4 (regulator of G-protein signaling 4): one of the regulatory proteins acting as GTPase activating proteins that drive G-alpha subunits into inactive GDP form, decreasing their activity. RGS4 inhibits Gq and Gi signaling. Reduced RGS4 leads to an excess of PKC signaling and impaired PFC cognitive function [228].
 DGKH (diacylglycerol kinase isoenzyme): one of the lipid kinases catalyzing conversion of diacylglycerol (DAG) to phosphatidic acid with an overall reduction in DAG which is a cofactor in the activation of PKC. Loss of DGKH leads to an increase in PKC signaling and mutations are linked to bipolar disease [229] and mania [230]. Of note is that the treatments with valproate, lithium, and tamoxifen inhibit PKC signaling [231].

### 8.2. Clinical Studies from Case Reports and Case Series of Animal and Human Data

*DLPFC Syndrome and Dopaminergic and Noradrenergic Therapy*. Using executive function tasks such as word fluency and trails tests, these have been associated with a positive response to clonidine in schizophrenia and Korsakoff's patients [232, 233]. Medications augmenting DA and NE systems have been shown to improve executive function in Tourette's syndrome and attention deficit hyperactivity disorder. A number of medications have been used including tricyclic antidepressants, guanfacine, clonidine, and deprenyl [234, 235]. In another example, idazoxan has been correlated with improved executive function in a frontotemporal lobe degeneration patient [236]. 

### 8.3. The AC Syndrome of Apathy and Akinetic Mutism

Based on animal and human data, dopamine agonists such as apomorphine and bromocriptine appear effective in treatment of the condition akinetic mutism spectrum of syndromes, for example. On the other hand, presynaptic dopaminergic agents (methylphenidate and carbidopa/levodopa) seem ineffectual [237–239]. Midbrain infarction with damage to the dopaminergic neurons also causes akinetic mutism and is responsive to DA agonists [240]. In patients where there is damage to the anterior cingulate gyrus with DA receptor damage, however, it has been speculated that DA agonists may fail. Hence loss of dopaminergic input from cortical structures such as the anterior cingulate gyrus as opposed to the striatum may be a factor in determining the type of DA treatment. However, other clinical studies in patients with apathy various psychiatric conditions, stroke, Wilson's disease, and HIV dementia have revealed benefit from array of DA-ergic medications including bromocriptine, amantadine, methylphenidate, buproprion, and selegiline [241–245].

### 8.4. Medial Orbitofrontal Syndrome, Disinhibition, and Behavioral Abnormalities Serotonergic Agents, Serenics, and Some AED's May Be Beneficial

Behavioral disinhibition correlates with a central serotonergic deficiency [246] and serotonergic treatment has been reported to be beneficial in treatment of aggression. One hypothesis regarding aggression is that it may be due to a downregulation of 5-HT2 receptors in the striatum and nucleus accumbens where they occur in abundance [247, 248]. Serotonergics such as fluoxetine and clomipramine may also be useful in disinhibited, impulsive, aggressive behavior [249]. Serenics (5HT 1A agonists) that bind to postsynaptic 5HT 1A receptors have been successful in treating aggression in animals. Propranolol, pindolol, and buspirone are examples [250]. In behavioral syndromes that may include mania and noradrenaline overactivity, adrenergic therapy may be beneficial in certain syndromes associated with bilateral inferior orbitofrontal contusions and respond to clonidine [251]. Other agents that have been useful include carbamazepine, sodium valproate, propranolol, clonidine, and lithium [252].

### 8.5. Lateral Orbitofrontal Syndrome and OCD

Aided recently by improved neuroimaging, namely, functional neuroimaging studies have delineated increased activity, either metabolism or blood flow in the orbitofrontal cortex, the head of caudate nucleus, and anterior cingulate gyrus [253]. In general, serotonin reuptake inhibitors as well as clomipramine have been the most advocated pharmacotherapeutic approaches, and right caudate head glucose metabolism (PET brain scan) was reduced with successful fluoxetine therapy for obsessive compulsive disorder (OCD) [254]. The interaction in cerebral DA and 5 HT may account for the improvement seen in some OCD with neuroleptics [255]. In cases where hypometabolism (PET brain scan) occurs in the anterior cingulate region and right OFC, this too has been correlated with an improved response to clomipramine therapy [256]. In refractory cases, sumatriptan (5HT 1D agonist) has improved both depression and OCD [257]. Both cognitive behavioral therapy and SSRI therapy have been shown effective in OCD treatment and their combination potentiated [258].

### 8.6. Randomized Controlled Trials: Human Studies

#### 8.6.1. Amantadine and Severe TBI

In a landmark international study, randomized, double-blinded, placebo-controlled trial of inpatient rehabilitation patients (*n* = 184) with minimally conscious or vegetative state were given amantadine 100 mg bid and increased to 200 mg bid by week 4. Outcome determined by the disability rating scale (DRS) and recovery was faster in the amantadine group as recorded by 0.24 units difference in the DRS per week over the period week 4 to week 16, in the DRS (*P* = 0.007). With discontinuation of amantadine, there was loss of function. The beneficial effects of amantadine were attributed to presynaptic release facilitation and postsynaptic reuptake blockade thereby augmenting dopaminergic transmission in the mesolimbic, nigrostriatal, and mesocortical circuitry that subserve attention, conation, and arousal [259].

#### 8.6.2. Methylphenidate and Moderate-to-Severe TBI

In one of the few randomized-controlled trials, the core frontal component of attention was found to significantly improve speed of information processing in 40 participants with moderate-to-severe TBI receiving methylphenidate at a dose of 0.3 mg/kg twice daily [260].

#### 8.6.3. Trazodone and Frontotemporal Lobe Disorder

In a meta-analysis, FTLD patients were presumed to have predominant serotonergic deficit as well as dopaminergic deficit with little evidence for Ach and NE related impairment. A double blinded, placebo-controlled, crossover trial of trazodone with 300 mg daily revealed a significant improvement using the neuropsychiatric inventory score. Trazodone is a selective serotonin reuptake inhibitor with SSRI, a 5HT1A, 5HT1C, and 5HT2, with the active metabolite being a direct serotonin receptor agonist as well as a adrenergic (alpha 1, alpha 2) and histamine (H1) blocking agent. The effects were noted in the domains of behavior rather than cognition [261, 262].

#### 8.6.4. Serotonergic Therapy and Stroke (Motor Deficit)

In the FLAME (fluoxetine for motor recovery after acute ischemic stroke) trial patients with ischemic stroke and moderate-to-severe motor deficit, the early use of fluoxetine in combination with physiotherapy enhanced motor recovery at a 3 month evaluation. The mechanism of action is suggested to be a modulation of spontaneous brain plasticity by drugs attributed to brain-derived neurotrophic factor [263].

#### 8.6.5. Neuropsychiatric Component Treatment

Partly preempted by the advent of the new DSM-V criteria, there is increased emphasis toward a neurobiological models of disease, a renewed neuroimaging focus and using dimensional scales as opposed to categorical diagnoses only (DSM-IV R) on traditional neuropsychiatric conditions [264]. The neuropsychiatric disorders include a diverse collection of syndromes affecting behavior, emotion, executive function, and other core frontal network functions that primarily affect emotion, executive function, higher cognition and their circuitry. With a distinct paucity of biomarkers for these syndromes, similar to the approach in neurology, it seems essential to integrate basic neuroscience, neuro-genetics, epigenetics, and neuroradiology in order to establish a foundation for diagnostic based on pathophysiology and presumed etiology. The current psychiatric classifications (DSM IV) have had the effect of dichotomizing disease when they are better configured dimensional traits overlapping with normality which is also in accordance with the polygenic mode of inheritance.

Many patients diagnosed according to the in DSM IV receive multiple diagnoses that is termed comorbidity, probably reflective of a diagnostic artifact due to symptom splitting and lumping. Perhaps the true underlying neurobiological process is due to single pathophysiological entity. Neither do current psychiatric medications respect DSM-IV defined boundaries disorders. Both antipsychotic and antidepressant agents, for example, are used to treat many different psychiatric disorders [264, 265].

Thinking in terms of symptom clusters and the core components of the frontal subcortical network circuitry may help construct a more neurobiological and pathophysiological relevant approach. This does, however, combine neurological, cerebrovascular, psychiatric, neuropsychological, general medical, and neuroradiological information and consequently the disciplines to advantage (Table 18).

### 8.7. Depression

Serotonergic agents, electroconvulsive therapy, transcranial magnetic stimulation physical exercise, and cognitive behavioral therapy have all been shown to benefit major depressive disorder [266–268]. An important study using PET brain scanning before and after treatment with CBT and a comparison group with the serotonergic agent paroxetine revealed changes in brain metabolism. The CBT group had increased activity in the hippocampal and dorsal cingulum and decreased in the frontal cortex activity whereas the paroxetine group had increased metabolism in the prefrontal cortex and decreased activity in the subgenual cingulate and hippocampus. These findings have been interpreted to suggest that CBT has a top down effect on the medial frontal and cingulate cortex and the pharmacological group work in a bottom up manner [269, 270]. Interpersonal psychotherapy (ITP) compared to venlafaxine similarly showed activation of the right posterior cingulate and right basal ganglia and of the right posterior temporal lobe and right basal ganglia in the venlafaxine group; this time assessed by SPECT scans [271]. Similarly, the psychosurgical treatment, namely, anterior cingulotomy, reserved for severe treatment resistant depression revealed a decrease of metabolism measured by PET brain scanning in the left subgenual PFC and left posterior thalamus, from the preoperative values [272].

### 8.8. Attention Deficit Hyperactivity Disorder

Stimulants such as methylphenidate increase NE and to a lesser extent DA in the PFC while producing lesser effects in the subcortical regions. Atomoxetine also increases NE and DA with less effect on striatal DA and may have a beneficial effect on impulsivity as well. Atomoxetine is an important new treatment option for adults with ADHD and is particularly so for those who are at risk for substance abuse. Atomoxetine is effective and a well-tolerated nonstimulant and the first ADHD treatment approved specifically for adult use administered as a single daily dose and is not a controlled substance. [273].

### 8.9. Emotional Component Treatment in Relation to Neurological or Psychiatric Conditions

Involuntary emotional expression disorder (IEED) is a more frequently diagnosed condition especially poststroke, traumatic brain injury, multiple sclerosis, and neurodegenerative diseases. Recently, pharmacological treatment has been successfully demonstrated with the efficacy of the dextromethorphan-quinidine combination (Nuedexta) [274, 275]. There are also behavioral programs that endeavor to improve one's emotional responses. Although in its infancy, one example of a regimen to improve one's emotional style comprising outlook, self-awareness, attention, resilience, social intuition, and sensitivity to context has been detailed by Davidson and Begley [276–278].

### 8.10. Task-Orientated and Repetitive Training-Based Interventions

Currently there is support for aphasia therapy, attentional training, rehabilitation of unilateral spatial neglect, and compensatory strategies for apraxia. Using information from randomized controlled trials, case series and single case reports to classify recommendations for the following forms of cognitive rehabilitation modalities has been made. 

#### 8.10.1. Randomized Controlled Trials

 Attention postTBI-attentional training—improvement
 Apraxia-apraxia training—improvement

#### 8.10.2. Systematic Reviews

Aphasia—intensive treatment. Improvement based on systemic reviews. 

Neglect poststroke—visual scanning and visuospatial motor training.

Attention disorders poststroke—attention task improvement.

Memory poststroke—errorless learning (electronic aids) —improvement [279].

#### 8.10.3. Constraint Therapy

This rationale of this mode of therapy proposes that in some patients there is a kind of learned nonuse of the paretic or paralyzed hand or arm after stroke or TBI. Physical therapy is applied to this limb while the unaffected limb is deliberately restrained. A phase III trial of constraint therapy of 2 weeks of such therapy resulted in significant gains that endured for approximately 2 years [280]. This was subsequently analyzed further to report that comparing early (3–9 months) and later (15–21 months) initiation of CIMT after stroke resulted in both groups achieving similar level of significant arm motor function 24 months after enrollment [281].

### 8.11. Devices

#### 8.11.1. Transcranial Magnetic Stimulation (TMS)

Depending on the pulse frequency, either hypofunction or improved function may ensue due to the inhibitory or excitatory effects on cortical function. This may have application in those instances where inhibitory cortical circuits are operative and if diminished function may return. A randomized trial of treatment-resistant depression with TMS has established this as a therapeutic component [282].

#### 8.11.2. Mirror Visual Feedback Therapy (MVF)

In controlled case series studies, MVF has been effective in treating poststroke paresis (arm or leg), phantom limb pain, and complex regional pain syndromeand anxiety. There is also evidence that the modality can modulate pain and reverse objective signs such as inflammation and paralysis [283, 284]. Both arm and leg paresis have been studied after stroke. Patients with arm paresis treated with MVF (*n* = 17, controls *n* = 19) with significant values were reported for hand FIM and arm and Brunnstrom scores at baseline, 4 weeks and at 6 months [285]. In a study of 40 patients with leg weakness after stroke, compared to best rehabilitation therapy and placebo-controlled with opaque glass, a statistically significant improvement was documented. Proposed postulated mechanisms included visuomotor tract restoration leading to an “unlearning of the learned paralysis” component after stroke [286]. This mechanism may also be attributed to a function of the mirror neuron system that involve interactions between the motor, vision, and proprioception modalities. Limb weakness after stroke may be both fiber tract damage, as well as-so-called learned paralysis whereby neurons and their fiber tracts are inhibited and that this can be unlearned using a mirror [287].

### 8.12. Mirror Neuron Therapy and Rehabilitation

Also called action observation treatment, this therapy is based on the premise that circuits are activated by observation, similar to those that perform the movement. Initial results from observational studies as an add-on therapy appear promising [287, 288].

## Figures and Tables

**Table 1:** Summary of cognitive psychological and neuroarcheological changes including brain size, reorganization, and NT changes.

Frontal lobe size as expected within hominoid evolution	
Frontoparietal sensory motor integration including mirror neuron circuitry	
Lunate sulcus moves more posteriorly with reduction in primary visual cortex	
Petalias left occipital, right frontal (cerebral torque)	
Neuropil less dense	
BA 10 increased	
BA 13 decreased	
Temporal lobe increased in size	
Amygdala nucleus increased	

**Table 2:** Core and extended emotional brain circuitry components.

Core emotional brain	
OFC: orbitofrontal cortex	
VMPFC: ventromedial prefrontal cortex	
ACC: anterior cingulate cortex	
BF: basal forebrain	
NA: nucleus accumbens	
Extended emotional brain	
PAG: periaqueductal gray matter	
ATL: anterior temporal lobe	
AI: anterior insula	
PCC: posterior cingulate cortex	
VTA: ventral tegmental area	

**Table 3:** Presentations of frontal network syndromes; clinical and radiological syndromes (Figure 10).

(A) Lesion studies (multimodality MRI or CT imaging)	
(1) Symptom related: most conditions present with the triad of inattention, executive dysfunction, and dysmemory. A working memory disorder (worried well) as opposed to early Alzheimer's disease is also frequent.	
(2) Syndrome related: basic clinical (abulia, disinhibition, dysexecutive).	
(3) Syndrome pathophysiologically related. Examples include frontal stroke, herpes simplex encephalitis, leukoaraiosis, watershed infarction such as “Man-in-the-Barrel syndrome”, or tumor related such as the Foster Kennedy syndrome.	
(4) Anatomically lobar: motor, premotor prefrontal dorsolateral, prefrontal mediobasal, and prefrontal orbitofrontal.	
(5) Anatomically network: frontal subcortical circuits	
(6) Anatomically long range network: brainstem, cerebellar, occipital lesions associated with FNS	
(B) No radiological abnormality-neurotransmitter syndromes	
Serotonin syndrome	
Neuroleptic malignant syndrome	
Malignant hyperpyrexia	
Cholinergic and anticholinergic toxidromes	
Paroxysmal autonomic instability and dystonia syndrome (PAIDS)	
(C) Synaptopathies (for example Limbic encephalitis)	
Disorders with antibodies against synaptic proteins such as NMDA, AMPA, and GABA-B receptors. Present with seizures and encephalopathies and yet are treatable [19].	
(D) Networktopathies and participatory networks (f-MRI)	
The default mode network, salience network, and attentional network may be evaluated by f-MRI (e.g., abnormal in AD, FTD, TBI, MS, depression, e.g.,) [20]	
Functional MRI-task-related activity seen, for example, with the Stroop, Word List Generation tests, and Wisconsin Card Sorting Test activating particular networks [21].	

**Table 4:** The more common clinical disorders presenting with neurological and/or psychiatric FNS.

(I) Neurological	
(a) Neurodegenerative	
Frontotemporal disorders (FTLD)	
Alzheimer's disease (AD)	
Cognitive vascular disorders (CVD)	
Frontal variant of AD	
Corticobasal-ganglionic disorders (CBD)	
(b) Cerebrovascular and cognitive vascular disorders	
Bland infarcts	
Strategic infarct	
Subcortical infarct	
Watershed infarct	
Frontal, sometimes bilateral as with common origin of both anterior cerebral arteries off the anterior communicating artery	
Leukoaraiosis	
Brainstem infarct	
Cerebellar infarct	
Strategic infarct such as caudate nucleus, basal ganglia, and thalamus	
Frontal lobe amyloid angiopathy	
Hemorrhage	
Amyloid angiopathy	
Microhemorrhage	
Subcortical hypertensive related	
(c) Tumors	
Frontal lobe meningioma (Foster Kennedy syndrome)	
(d) Traumatic brain injury	
Diffuse axonal injury	
Chronic subacute encephalopathy	
(e) Multiple sclerosis	
(f) Parkinson's, Huntington's	
(g) Frontal lobe epilepsies	
(h) Normal pressure hydrocephalus	
(i) Neurotoxicology—alcohol	

(II) Psychiatric	

Schizophrenia	
Mania and hypomania	
Depression	
Anxiety	
Obsessive compulsive	
Tourette's	
Attention deficit hyperactivity disorder (ADHD)	
Autism	
William's syndrome	
Pervasive developmental disorders	

**Table 5:** Additional neuropathological states and conditions in which FNS is invariably part of the neurological syndrome [22–24].

Subcortical gray matter	
HIV dementia	
Wilson's disease	
Huntington's	
Neuroacanthocytosis syndrome	
Prionopathies (Creutzfeldt-Jakob, GSS FFI, and BSE)	
Fahr's syndrome—calcification of the BG	
Pantothenate kinase 2 associated neurodegeneration (PANK2)	
Adult neuronal ceroid lipofuscinosis	
Subcortical white matter	
Leukodystrophy disorders (metachromatic, Krabbe's, adrenal, orthochromatic)	
Fabry's disease	
Vanishing white matter disease	
Mixed cortical and subcortical pathology	
Vasculitides	
Meningitis/encephalitis	
CADASIL	
Alexander's disease	
Canavan disease	
Cerebrotendinous xanthomatosis	
Polycystic lipomembranous osteodysplasia with sclerosing leukoencephalopathy (PLOSL) or Nasu-Hakola disease	
Mitochondrial diseases (MELAS, MERFF, and Kearns Sayre)	

**Table 6:** Dementias have clinical pathological and molecular components.

Domain	Pathology	Clinical	Subtypes
Linguistic	Tauopathies	Picks, SD, PPA.	3
Comportmental	Tauopathies	FTD behavioral	1
Amnestic	Amyloidopathies	AD	4
Movement dis.	Synucleinopathies	PD, DLB, PSP, CBD	5

**Table 7:** Neuropathologic subtypes.

Histopathology	Subtypes	Clinical	Chromosome
Tauoapthy	PiD	bvFTD, PNFA	17 (MAPT)
	PSP	PSP	
	CBD	CBD, PNFA	
	AGD	bvFTD, MND	
	MST		

TDP-43	Type 1	bvFTD	
	Type 2	SD, MND	9 (IFT74)
	Type 3	bvFTD, SD, PNFA	17 (PGRN)
	Type 4	bvFTD, myopathy, Paget's	9 (VCP)

FTLD-UPS	FTD 3	bvFTD	3 (CHMP2B)

BIBD	—	FTD-MND	

FTLD-IF	—	—	—

FTLD-ni	—	—	—

PiD: Pick's disease, PSP: progressive supranuclear palsy, CBD: cortico-basal-ganglionic degeneration, AGD: argyrophilic grain disease, MND: motor neuron disease, PNFA: progressive nonfluent aphasia, SD: semantic disease/variant, MST: sporadic multisystem tauopathy, BIBD: basophilic inclusion body disease, FTLD IF: FTLD with intermediate filament inclusions, FTLD ni: FTLD with no inclusions, FUS: fused in sarcoma, NIF: neuronal intermediate filaments, VCP: valosin containing protein, CMBP 2B: charged multivesicular body protein 2B, MAPT: microtubule associated protein tau, TDP-43: TAR DNA binding protein 43, IFT74: intraflagellar transport protein 74.

Modified from Josephs [25].

**Table 8:** Conditions that can present with white matter hypertensities on MRI brain scan.

(i) Cerebrovascular (HTN, Atrial fibrillation, DM, Homocysteine)	
(ii) Alzheimer's	
(iii) APOE 4 status	
(iv) Trauma	
(v) Migraine	
(vi) AIDS dementia	
(vii) Psychiatric (bipolar, schizophrenia)	
(viii) Autism	
(ix) CADASIL	
(x) Wilson's, Hallervorden Spatz	
(xi) Dystonia	
(xii) Neuroacanthocytosis	
(xiii) Fragile X associated tremor and ataxia	
(xiv) Susac's syndrome	
(xv) Myotonic dystrophy type 1 and 2	
(xvi) Hypoglycemic encephalopathy	
(xvii) Leukodystrophies (Metachromatic, Krabbe)	
(xviii) Multiple sclerosis	
(xix) Autoimmune vasculitis (SLE, Sjogren's)	

**Table 9:** Junque Leukoaraiosis grading [26].

Evaluate 5 areas in each hemisphere	
(i) Centrum semiovale in frontal region	
(ii) Centrum semiovale parietal region	
(iii) White matter surrounding frontal horn	
(iv) White matter surrounding corpus of the lateral ventricle	
(v) White matter surrounding the atrium and occipital horn of lateral ventricle	

Numerical scores 1–4 of T2 hyperintensity	
(i) No changes	0
(ii) <25%	1
(iii) 25%–50%	2
(iv) 50%–75%	3
(v) >75%	4

Total score	0–40

**Table 10:** Pathophysiology of concussion and traumatic brain injury [27, 28].

(1) Excessive or indiscriminate release of excitatory neurotransmitters	
Increased glutamate binding to NMDA receptors causes efflux of potassium out of the cell, influx of calcium, and alteration of the neuronal membrane potential: Na-K pump is upregulated and consequently requires more ATP.	
(2) An uncoupling of glucose metabolism and cerebral blood flow occurs	
A glucose hypermetabolism ensues and there is a simultaneous diminished cerebral blood flow, which may be reduced as much as 50% of normal	
Even more important from a clinical point of view, the cerebral glucose may be reduced for up to 4 weeks (measured by PET brain scan in humans)	
(3) Calcium accumulation occurs	
Intracellular Ca^++^ accumulation causes mitochondrial impairment, cell death by phosphokinases, protein kinases, NO synthase, endonucleases, and calpains and plasmalogenase culminating in free radical accumulation and apoptosis.	
(4) Chronic alterations in neurotransmission	
Glutaminergic, cholinergic, and adrenergic alterations account for the memory and cognitive deficits seen after concussion and TBI. The neurochemical findings include LTP may be persistently impaired after TBI, loss of cholinergic input from the basal forebrain, and impaired GABA inhibitory function of the hippocampal dentate granule cells occurs which predisposes the injured brain to seizures.	
(5) Axonal disconnection occurs	
Diffuse axonal injury may occur due to mechanical stretching or calcium influx with subsequent microtubule breakdown. Axonal bulbs may result due to intra-axonal cytoskeletal injury, accumulation of organelles at the site of damage axonal damage with localized axonal swellings appearing (axonal bulbs). Secondary axonotomy (constrictions) with axonal disconnection may occur many weeks after TBI.	

**Table 11:** Different classification systems and current status of antibodies implicated: autoimmune dementia may be idiopathic or secondary to cancer (paraneoplastic) [29].

Eponymous	
Morvan syndrome	
Syndromic	
Progressive encephalomyelopathy with rigidity and myoclonus	
Serologically	
VGKC antibody associated encephalopathy	
Pathologically	
Nonvasculitic autoimmune meningoencephalitis	
Antibodies	
VGKC	
NMDA receptor antibody	
AMPA receptor	
GABA_B_ receptor	
GAD 65	
ANNA-1 (anti-Hu)	
ANNA-2 (anti-Ri)	
ANNA-3	
AGNA (SOX-1)	
PCA-2	
CRMP-5 (anti-CV2)	
Amphiphysin	
Ma/Ta proteins	
NMO-IgG	

VGKC: voltage gated potassium channel, NMDA: N-methyl D-aspartate, AMPA: alpha-amino-3-hydroxy-5-methyl-isoxazolepropionic acid, GABA: gamma-aminobutyric acid, GAD: glutamic acid decarboxylase 65, ANNA, antineuronal nuclear antibody, AGNA: antiglial nuclear antibody, PCA: Purkinje cell cytoplasmic antibody, CRMP 5: collapsing response mediator protein 5, NMO: neuromyelitis optica IgG antibodies (modified from Mckeon et al.) [30].

**Table 12:** Viral, bacterial, fungal, and parasitic brain infections with frontal subcortical circuit involvement.

Viral	
HIV encephalopathy	
JC virus—progressive multifocal leukoencephalopathy (PML)	
Herpes simplex encephalitis	
West Nile virus	
Tegmentothalamic syndrome (various)	
Bacterial	
Tuberculous meningitis	
Neisseria meningitides	
Hemophilus influenza	
Listeria monocytogenes	
Whipple's disease	
Spirochetal	
Borreliosis (Lyme disease)	
Neurolues	
Fungal	
Cryptococcal meningitis	
Histoplasmosis	
*Coccidioides immitis *	
*Blastomyces dermatitidis *	
Candida species	
Prionopathies	
Creutzfeldt Jakob disease (CJD)	
Variant Creutzfeldt-Jakob disease (V-CJD)	
Kuru	
Fatal familial insomnia (FFI)	
Gerstmann-Straeussler-Scheinker syndrome (GSS)	
Parasitic	
Malaria	
Bilharziasis	
Cysticercosis	
Toxoplasmosis	
Amoebic meningitis (*Naegleri Fowleri*)	

**Table 13:** Cerebral vasculitides and autoimmune disorders classification.

Primary	
Polyarteritis nodosa	
Cogan's syndrome	
Churg-Strauss syndrome	
Temporal	
Takayasu's Disease	
Granulomatous	
Lymphomatoid	
Wegener's	
Kawasaki	
Susac's	
Hypersensitivity	
Buergers	
Acute posterior multifocal placoid pigment epitheliopathy	
Kohlmeier Degos	
Isolated angiitis of the CNS	
Secondary to autoimmune and systemic diseases	
Sarcoidosis	
Rheumatoid arthritis	
Systemic lupus erythematosus	
Sjogrens	
Behcet's	
Scleroderma	
Mixed connective tissue disease	
Dermatomyositis	
Ulcerative colitis	
Coeliac disease	
Secondary—infectious related	
Human immunodeficiency virus	
Varicella zoster	
Herpes zoster	
Cytomegalovirus	
Mycotic	
Lues disease	
*Borrelia burgdorferi *	
Tuberculosis	
Cysticercosis	
Bacterial meningitis	
Secondary to neoplasia	
Hodgkins and non-Hodgkins	
Malignant histiocytosis	
Hairy cell leukemia	
Secondary to illicit drugs	
Cocaine	
Sympathomimetic agents	
Amphetamine	

**Table 14:** Anatomical and functional imaging categories and examples of major disease entities associated with frontal network syndromes [31–34].

(A) MRI multimodality	
MRI (routine series)	
T1/T2, FLAIR, GRE, and MRA to detect degree of concomitant vascular disease, atrophy pattern, and other secondary pathologies	
MRI—DTI	
Fiber tract pathology especially in traumatic brain injury, multiple sclerosis	
MRI quantitative atrophy estimation	
Different patterns of the major dementia syndromes (Seeley et al. [20])	
MRI—perfusion	
Perfusion as a reflection of hypometabolism, similar to SPECT (perfusion) and PET (metabolism) patterns of abnormality	
MR spectroscopy	
Biochemical analysis of NAA, choline, lactate particularly useful in brain tumor diagnosis	
(B) SPECT	
Hypoperfusion (in vascular or hypometabolism)	
Hyperperfusion for example with ictal foci.	
(C) PET brain	
Hypometabolic patterns in different dementias	
(D) Intrinsic state connectivity maps	
Default mode	
Salience network	
Attentional network	
Visual network	
Auditory network	
(E) Quantitative EEG and MEG	
AD reduced connectivity of alpha and beta in frontoparietal and frontotemporal regions	
Parkinson's increased connectivity of alpha and beta locally and globally	
DLBD reduced connectivity alpha range locally and globally.	

Adapted and modified from [31, 32].

**Table 15:** PET brain patterns in dementias.

Dementia subtype	^ 18 F^FDG PET hypometabolism pattern
Alzheimer	Relatively symmetric parietotemporal, medial temporal, posterior cingulate, and frontal association cortex to lesser degree
AD variant (PCAS)	Occipital hypometabolism predominates
FTD behavioral variant	Frontal and anterior temporal hypometabolism
PDD	Temporoparietal, may be similar to AD
DLBD	Occipital and temporal hypometabolism
CVD	Cortical and subcortical, singular or multifocal, correlating with structural imaging abnormality
CBD	Global reduction in metabolism as well as asymmetric prefrontal, premotor, sensorimotor superior temporal, parietal hypometabolism with thalamic hypometabolism contralateral to limb apraxia
Huntington's	Caudate nucleus hypometabolism and frontal association cortex to a lesser degree
PSP	Caudate nucleus, putamen, thalamus, pons, and superior and anterior frontal cortex

PCAS: posterior cortical atrophy syndrome.

PSP: progressive supranuclear palsy.

FDG PET increases diagnostic accuracy beyond that derived from clinical evaluation. Adapted and modified from [35].

**Table 16:** Intrinsic connectivity network patterns in dementias.

Dementia subtype	Intrinsic connectivity pattern
Alzheimer	Default mode network shows reduced connectivity
FTD behavioral variant	Salience network shows reduced connectivity
Parkinson's	BN-thalamocortical loops show increased connectivity
DLBD	Uncertain at present but may show ascending brainstem projection system
CBG	Uncertain

FTD: frontotemporal lobe disorder, DLDB: diffuse lewy body disease, BN: basal nuclei (basal ganglia), CBG: corticobasal-ganglionic disorder.

Adapted and modified from [35].

**Table 17:** Future treatment strategies proposed for the stroke model.

(1) Small molecules (monoaminergic systems, antibodies against axonal growth inhibitor Nogo-A)	
(2) Growth factors (fibroblast growth factor, brain derived neurotrophic factor, hematopoietic growth factor, granulocyte colony stimulating factor)	
(3) Cell-based therapies (endothelial progenitor cells, intracerebral transplantation of cultured neuronal cells, intravenous mesenchymal stromal cells)	
(4) Electromagnetic stimulation	
(5) Device-based therapies	
(6) Task orientated and repetitive training-based interventions	

Modified from [36].

**Table 18:** Proposed approach of neurological/neuropsychiatric disorders.

(1) Use the list of symptoms and signs to form an overall generic categorical diagnostic syndromes such as abulic/apathetic, disinhibited/dysexecutive, depression, and obsessive compulsive disorders. This is a clinical assessment that may be aided by inventories, scales, or diagnostic manuals such as DSM IV	
(2) Component analysis in terms of the core frontal functions embedded in the 5 currently appreciated frontal subcortical behavioral circuits	
(3) Establish the cerebrovascular component and its specific treatment	
(4) Establish whether medical conditions (hypothyroidism, low B12, Vit D, folate) are contributing to the cognitive impairment	
(5) Establish contribution of impaired sleep (sleep apnea, dyssomnia)	
(6) Establish contribution of centrally acting drugs and discontinue, reduce dosage or change to another less conflicting drug if possible.	
(7) Use known information about neurotransmitter deficiencies in these syndromes and/or FSC's and target with specific pharmacological and behavioral treatment approaches.	
(8) Counsel with respect to the 5 principal components of brain health	

## References

[1] Karussis D, Leker RR, Abramsky O (2000). Cognitive dysfunction following thalamic stroke: a study of 16 cases and review of the literature. *Journal of the Neurological Sciences*.

[2] Kumral E, Evyapan D, Balkir K (1999). Acute caudate vascular lesions. *Stroke*.

[3] Tullberg M, Fletcher E, DeCarli C (2004). White matter lesions impair frontal lobe function regardless of their location. *Neurology*.

[4] Neau JP, Anllo EA, Bonnaud V, Ingrand P, Gil R (2000). Neuropsychological disturbances in cerebellar infarcts. *Acta Neurologica Scandinavica*.

[5] Hoffmann M, Schmitt F (2004). Cognitive impairment in isolated subtentorial stroke. *Acta Neurologica Scandinavica*.

[6] Malm J, Kristensen B, Karlsson T, Carlberg B, Fagerlund M, Olsson T (1998). Cognitive impairment in young adults with infratentorial infarcts. *Neurology*.

[7] Garrard P, Bradshaw D, Jäger HR, Thompson AJ, Losseff N, Playford D (2002). Cognitive dysfunction after isolated brain stem insult. An underdiagnosed cause of long term morbidity. *Journal of Neurology Neurosurgery and Psychiatry*.

[8] Goldberg E (2001). *The Executive Brain: Frontal Lobes and the Civilized Mind*.

[9] Chow TW, Cummings JL, Miller B, Cummings JL (2009). Frontal subcortical circuits. *The Human Frontal Lobes*.

[10] Lichter DG, Cummings JL (2001). *Frontal Subcortical Circuits in Psychiatric and Neurological Disorders*.

[11] Dobzhansky T (1973). Nothing in biology makes sense except in the light of evolution. *American Biology Teacher*.

[12] Dawkins R (2004). *The Ancestor’s Tale. A Pilgrimage to the Dawn of Evolution*.

[13] Zalc B, Goujet D, Colman DR (2008). The origin of the myelination program in vertebrates. *Current Biology*.

[14] Roots BI (1993). The evolution of myelin. *Advances in Neural Science*.

[15] Coppens Y (1994). East side story: the origin of humankind. *Scientific American*.

[16] Marean CW (2010). When the sea saved humanity. *Scientific American*.

[17] Delange F (2000). The role of iodine in brain development. *Proceedings of the Nutrition Society*.

[18] Wainwright PE (2002). Dietary essential fatty acids and brain function: a developmental perspective on mechanisms. *Proceedings of the Nutrition Society*.

[19] Kelley BJ, Boeve BF, Josephs KA (2008). Young-onset dementia: demographic and etiologic characteristics of 235 patients. *Archives of Neurology*.

[20] Seeley WW, Menon V, Schatzberg AF (2007). Dissociable intrinsic connectivity networks for salience processing and executive control. *Journal of Neuroscience*.

[21] Fuster JM, Fuster JM (2009). Neuroimaging. *The Prefrontal Cortex*.

[22] Prayson RA, Goldblum JR (2005). *Neuropathology*.

[23] Brunnström H, Gustafson L, Passant U, Englund E (2009). Prevalence of dementia subtypes: a 30-year retrospective survey of neuropathological reports. *Archives of Gerontology and Geriatrics*.

[24] Mercy L, Hodges JR, Dawson K, Barker RA, Brayne C (2008). Incidence of early-onset dementias in Cambridgeshire, United Kingdom. *Neurology*.

[25] Josephs KA (2008). Frontotemporal dementia and related disorders: deciphering the enigma. *Annals of Neurology*.

[26] Junque C, Pujol J, Vendrell P (1990). Leuko-araiosis on magnetic resonance imaging and speed of mental processing. *Archives of Neurology*.

[27] Giza CC, Hovda DA (2001). The neurometabolic cascade of concussion. *Journal of Athletic Training*.

[28] Barkhoudarian G, Hovda DA, Giza CC (2011). The molecular pathophysiology of concussive brain injury. *Clinics in Sports Medicine*.

[29] Vernino S, Geschwind M, Boeve B (2007). Autoimmune encephalopathies. *Neurologist*.

[30] McKeon A, Lennon VA, Pittock SJ (2010). Immunotherapyresponsive dementias and encephalopathies. *Continuum Lifelong Learning in Neurology*.

[31] Standley K, Brock C, Hoffmann M (2012). Advances in functional neuroimaging in dementia and potential pitfalls. *Neurology International*.

[32] Small SA, Schobel SA, Buxton RB, Witter MR, Barnes CA (2011). A pathophysiological framework of hippocampal dysfunction in ageing and disease. *Nature Reviews Neuroscience*.

[33] Petrella JR, Sheldon FC, Prince SE, Calhoun VD, Doraiswamy PM (2011). Default mode network connectivity in stable vs progressive mild cognitive impairment. *Neurology*.

[34] Zeng L, Shen H, Liu L (2012). Identifying major depression using whole brain functional connectivity a multivariate pattern analysis. *Brain*.

[35] Seeley WW, Menon V, Schatzberg AF (2007). Dissociable intrinsic connectivity networks for salience processing and executive control. *Journal of Neuroscience*.

[36] Cramer SC (2008). Repairing the human brain after stroke. II. Restorative therapies. *Annals of Neurology*.

[37] Klein RG (2009). *The Human Career: Human Biological and Cultural Origins*.

[38] Holloway RL (1983). Human brain evolution: a search for units, models and synthesis. *Canadian Journal of Anthropology*.

[39] Semendeferi K, Damasio H, Frank R, van Hoesen GW (1997). The evolution of the frontal lobes: a volumetric analysis based on three-dimensional reconstructions of magnetic resonance scans of human and ape brains. *Journal of Human Evolution*.

[40] Semendeferi K, Lu A, Schenker N, Damasio H (2002). Humans and great apes share a large frontal cortex. *Nature Neuroscience*.

[41] Holloway RL, Broadfield D, Yuan M, Schick K, Toth N (2010). The human brain evolving. A personal perspective. *The Human Brain Evolving*.

[42] Semendeferi K, Armstrong E, Schleicher A, Zilles K, van Hoesen GW (1998). Limbic frontal cortex in hominoids: a comparative study of area 13. *American Journal of Physical Anthropology*.

[43] Hof PR, Mufson EJ, Morrison JH (1995). Human orbitofrontal cortex: cytoarchitecture and quantitative immunohistochemical parcellation. *Journal of Comparative Neurology*.

[44] Hof PR, van der Gucht E (2007). Structure of the cerebral cortex of the humpback whale, Megaptera novaeangliae (Cetacea, Mysticeti, Balaenopteridae). *Anatomical Record*.

[45] Semendeferi K, Armstrong E, Schleicher A, Zilles K, van Hoesen GW (2001). Prefrontal cortex in humans and apes: a comparative study of area 10. *American Journal of Physical Anthrorpology*.

[46] Schenker NM, Desgouttes AM, Semendeferi K (2005). Neural connectivity and cortical substrates of cognition in hominoids. *Journal of Human Evolution*.

[47] Schumann CM, Amaral DG (2005). Stereological estimation of the number of neurons in the human amygdaloid complex. *Journal of Comparative Neurology*.

[48] Barton RA, Aggleton JP, Grenyer R (2003). Evolutionary coherence of the mammalian amygdala. *Proceedings of the Royal Society B*.

[49] Brothers L (1990). The social brain: a project for integrationg primate behavior and neurophsyiology in a new domain. *Concepts in Neuroscience*.

[50] Barger N, Stefanacci L, Semendeferi K (2007). A comparative volumetric analysis of the amygdaloid complex and basolateral division in the human and ape brain. *American Journal of Physical Anthropology*.

[51] Previc FH (1999). Dopamine and the origins of human intelligence. *Brain and Cognition*.

[52] Bortz WM (1985). Physical exercise as an evolutionary force. *Journal of Human Evolution*.

[53] Carrier DR (1984). The energetic paradox of human running and hominid evolution. *Current Anthropology*.

[54] Leonard WR, Robertson MS (1997). Comparative primate energetics and hominid evolution. *American Journal of Physical Anthropology*.

[55] Raghanti MA, Stimpson CD, Marcinkiewicz JL, Erwin JM, Hof PR, Sherwood CC (2008). Cortical dopaminergic innervation among humans, chimpanzees, and macaque monkeys: a comparative study. *Neuroscience*.

[56] Berger B, Gaspar P, Verney C (1991). Dopaminergic innervation of the cerebral cortex: unexpected differences between rodents and primates. *Trends in Neurosciences*.

[57] Lewis DA, Melchitzky DS, Sesack SR, Whitehead RE, Auh S, Sampson A (2001). Dopamine transporter immunoreactivity in monkey cerebral cortex: regional, laminar, and ultrastructural localization. *Journal of Comparative Neurology*.

[58] Jakab RL, Goldman Rakic PS (2000). Segregation of serotonin 5HT 2A and 5HT 3 receptors in inhibitory circuits in the primate cerebral cortex. *The Journal of Comparative Neurology*.

[59] Soubrié P (1986). Reconciling the role of central serotonin neurons in human and animal behavior. *Behavioral and Brain Sciences*.

[60] Sarter M, Parikh V (2005). Choline transporters, cholinergic transmission and cognition. *Nature Reviews Neuroscience*.

[61] Levin ED, Simon BB (1998). Nicotinic acetylcholine involvement in cogitive function in animals. *Psychopharmacology*.

[62] Subiaul F, Broadfield D, Yuan M, Schick K, Toth N (2010). Mosaic cognitive evolution: the case of imitation behavior. *The Human Brain Evolving*.

[63] Brady ST, Siegel GJ, Albers RW, Price DL (2012). *Basic Neurochemistry: Principles of Molecular, Cellular and Medical Neurobiology*.

[64] Nestler EJ, Hyman SE, Malenka (2009). *Molecular Neuropharmacology: A Foundation for Clinical Neurocience*.

[65] Lichter DG, Cummings JL, Lichter DG, Cummings JL (2001). Introduction and overview. *Frontal—Subcortical Circuits in Psychiatric and Neurological Disorders*.

[66] Cummings JL (1993). Frontal-subcortical circuits and human behavior. *Archives of Neurology*.

[67] Catani M, Jones DK, Ffytche DH (2005). Perisylvian language networks of the human brain. *Annals of Neurology*.

[68] Mallet N, Micklem BR, Henny P (2012). Dichotomous organization of the external globus pallidus. *Neuron*.

[69] Yeterian EH, Pandya DN (1993). Striatal connections of the parietal association cortices in rhesus monkeys. *Journal of Comparative Neurology*.

[70] Stuss DT, Floden D, Alexander MP, Levine B, Katz D (2001). Stroop performance in focal lesion patients: dissociation of processes and frontal lobe lesion location. *Neuropsychologia*.

[71] Fesenmeier JT, Kuzniecky R, Garcia JH (1990). Akinetic mutism caused by bilateral anterior cerebral tuberculous obliterative arteritis. *Neurology*.

[72] Bogousslavsky J, Regli F (1990). Anterior cerebral artery territory infarction in the Lausanne stroke registry. *Archives of Neurology*.

[73] Berthier ML, Kulisevsky J, Gironell A, Heras JA (1996). Obsessive-compulsive disorder associated with brain lesions: clinical phenomenology, cognitive function, and anatomic correlates. *Neurology*.

[74] Berthier ML, Starkstein SE, Robinson RG, Leiguarda R (1990). Limbic lesions in a patient with recurrent mania. *Journal of Neuropsychiatry and Clinical Neurosciences*.

[75] Stuss DT, Miller B, Cummings JL (2009). New approaches to prefrontal lobe testing. *The Human Frontal Lobes*.

[76] Stuss DT, Binns MA, Murphy KJ, Alexander MP (2002). Dissociations within the anterior attentional system: effects of task complexity and irrelevant information on reaction time speed and accuracy. *Neuropsychology*.

[77] Stuss DT, Guberman A, Nelson R, Larochelle S (1988). The neuropsychology of paramedian thalamic infarction. *Brain and Cognition*.

[78] Cicerone KD (1996). Attention deficits and dual task demands after mild traumatic brain injury. *Brain Injury*.

[79] Aharon-Peretz J, Tomer R, Miller B, Cummings JL (2009). Traumatic brain injury. *The Human Frontal Lobes*.

[80] Bar-On R, Tranel D, Denburg NL, Bechara A (2003). Exploring the neurological substrate of emotional and social intelligence. *Brain*.

[81] Shamay-Tsoory SG, Tomer R, Goldsher D, Berger BD, Aharon-Peretz J (2004). Impairment in cognitive and affective empathy in patients with brain lesions: anatomical and cognitive correlates. *Journal of Clinical and Experimental Neuropsychology*.

[82] Pessoa L (2008). On the relationship between emotion and cognition. *Nature Reviews Neuroscience*.

[83] Hoffmann M, Benes Cases L, Hoffmann B, Chen R (2010). The impact of stroke on emotional intelligence. *BMC Neurology*.

[84] Kapur N (1996). Paradoxical functional facilitation in brain-behaviour research: a critical review. *Brain*.

[85] Schott GD (2012). Pictures as a neurological tool: lessons from enhanced and emergent artistry in brain disease. *Brain*.

[86] Christodoulou GN, Margariti M, Kontaxakis VP, Christodoulou NG (2009). The delusional misidentification syndromes: strange, fascinating, and instructive. *Current Psychiatry Reports*.

[87] Pell MD (2006). Judging emotion and attitudes from prosody following brain damage. *Progress in Brain Research*.

[88] Peña-Casanova J, Roig-Rovira T, Bermudez A, Tolosa-Sarro E (1985). Optic aphasia, optic apraxia, and loss of dreaming. *Brain and Language*.

[89] Treffert DA (2010). *Islands of Genius*.

[90] Assessment: neuropsychological testing of adults. Considerations for neurologists.

[91] Kramer JH, Reed BR, Mungas D, Weiner MW, Chui HC (2002). Executive dysfunction in subcortical ischaemic vascular disease. *Journal of Neurology Neurosurgery and Psychiatry*.

[92] Prins ND, van Dijk EJ, den Heijer T (2005). Cerebral small-vessel disease and decline in information processing speed, executive function and memory. *Brain*.

[93] Nasreddine ZS, Phillips NA, Bédirian V (2005). The montreal cognitive assessment, MoCA: s brief screening tool for mild cognitive impairment. *Journal of the American Geriatrics Society*.

[94] Dubois B, Slachevsky A, Litvan I, Pillon B (2000). The FAB: a frontal assessment battery at bedside. *Neurology*.

[95] Royall DR, Mahurin RK, Gray KF (1992). Bedside assessment of executive cognitive impairment: the executive interview. *Journal of the American Geriatrics Society*.

[96] Hoffmann M, Schmitt F, Bromley E (2009). Comprehensive cognitive neurological assessment in stroke. *Acta Neurologica Scandinavica*.

[97] Hoffmann M, Schmitt F (2006). Metacognition in stroke: bedside assessment and relation to location, size, and stroke severity. *Cognitive and Behavioral Neurology*.

[98] Dwolatzky T, Whitehead V, Doniger GM (2003). Validity of a novel computerized cognitive battery for mild cognitive impairment. *BMC Geriatrics*.

[99] Robbins TW, James M, Owen AM, Sahakian BJ, McInnes L, Rabbitt P (1994). Cambridge neuropsychological test automated battery (CANTAB): a factor analytic study of a large sample of normal elderly volunteers. *Dementia*.

[100] Kiernan RJ, Mueller J, Langston JW, van Dyke C (1987). The neurobehavioral cognitive status examination: a brief but differentiated approach to cognitive assessment. *Annals of Internal Medicine*.

[101] Gualtieri CT, Johnson LG (2006). Reliability and validity of a computerized neurocognitive test battery, CNS Vital Signs. *Archives of Clinical Neuropsychology*.

[102] Delis DC, Kaplan E, Kramer JH (2001). *DKEFS*.

[103] Wechsler D (2008). *Wechsler Adult Intelligence Scale*.

[104] Grace J, Malloy PF (2001). *Frontal Systems Behavior Scale*.

[105] Roth RM, Isquith PK, Gioia GA (2005). *BRIEF-A: Behavior Rating Inventory of Executive Funtion-Adult Version*.

[106] Kertesz A, Davidson W, Fox H (1997). Frontal behavioral inventory: diagnostic criteria for frontal lobe dementia. *Canadian Journal of Neurological Sciences*.

[107] Reynolds CR (2002). *Comprehensive Trail Making Test*.

[108] Gladsjo JA, Walden Miller W, Heaton RK (1999). *Norms for Letter and Category Fluency: Demographic Corrections for Age, Education and Ethnicity*.

[109] Heaton RK (2003). *Wisconsin Card Sorting Test Computer Version 4*.

[110] Culbertson WC, Zillmer EA (2001). *Tower of London*.

[111] Bar-On R (1997). *The Bar-On EmotIonal QuotIent Inventory (EQ-I): TechnIcal Manual*.

[112] MSCEIT (2002). *Mayer, Salovey, & Caruso*.

[113] Trenerry MR, Crosson B, DeBoe J, Leber WR (1989). *Stroop Neuropsychological Screening Test*.

[114] Bechara A (2007). *Iowa Gambling Test*.

[115] Rutter M, Le Couteur A, Lord C (2005). *ADI-R*.

[116] Baron-Cohen S, Wheelwright S, Skinner R, Martin J, Clubley E (2001). The autism-spectrum quotient (AQ): evidence from asperger syndrome/high-functioning autism, males and females, scientists and mathematicians. *Journal of Autism and Developmental Disorders*.

[117] Brown TE (1996). *Brown Attention Deficit Disorder Scales*.

[118] Radloff L (1977). The CES-D scale: a self report depression scale for research in the general population. *Applied Psychological Measurement*.

[119] Beck AT, Steer RA, Brown GK (1996). *Beck Depression Inventory II*.

[120] Reynolds WM, Kobak KA (1995). *Hamilton Depression Inventory*.

[121] Stone VE, Baron-Cohen S, Knight RT (1998). Frontal lobe contributions to theory of mind. *Journal of Cognitive Neuroscience*.

[122] Baron-Cohen S, Jolliffe T, Mortimore C, Robertson M (1997). Another advanced test of theory of mind: evidence from very high functioning adults with autism or Asperger syndrome. *Journal of Child Psychology and Psychiatry and Allied Disciplines*.

[123] Manly T, Hawkins K, Evans J, Woldt K, Robertson IH (2002). Rehabilitation of executive function: facilitation of effective goal management on complex tasks using periodic auditory alerts. *Neuropsychologia*.

[124] Knight C, Alderman N, Burgess PW (2002). Development of a simplified version of the multiple errrands test for use in hospital settings. *Neuropsychological Rehabilitation*.

[125] Windmann S, Wehrmann M, Calabrese P, Güntürkün O (2006). Role of the prefrontal cortex in attentional control over bistable vision. *Journal of Cognitive Neuroscience*.

[126] Torrance EP (1970). Influence of dyadic interaction on creative functioning. *Psychological Reports*.

[127] Trenerry MR, Cross B, de Boe J, Leber WR (1990). *Visual Search and Attention Test (VSAT)*.

[128] Bernstein JH, Waber DP (1996). *Developmental Scoring System for the Rey Osterrieth Complex Figure*.

[129] Goldberg E, Podelle K, Bilder R, Jaeger J (1999). *The Executive Control Battery*.

[130] Kertesz A (1982). *The Western Aphasia Battery*.

[131] Goodglass H, Kaplan E, Barresi B (2001). *Boston Diagnostic Aphasia Test*.

[132] Doty R (2006).

[133] Mesulam MM (1990). Large-scale neurocognitive networks and distributed processing for attention, language, and memory. *Annals of Neurology*.

[134] Goldberg E (2001). *The Executive Brain: Frontal Lobes and the Civilized Mind*.

[135] Winter B, Bert B, Fink H, Dirnagl U, Endres M (2004). Dysexecutive syndrome after mild cerebral ischemia? Mice learn normally but have deficits in strategy switching. *Stroke*.

[136] Gillman PK (2006). A review of serotonin toxicity data: implications for the mechanisms of antidepressant drug action. *Biological Psychiatry*.

[137] Gnanadesignan N, Espinoza RT, Smith RL (2005). The serotonin syndrome. *The New England Journal of Medicine*.

[138] Whyte IM (2004). *Serotonin Toxicity/Syndrome: Medical Toxicology*.

[139] Strawn JR, Keck PE, Caroff SN (2007). Neuroleptic malignant syndrome. *American Journal of Psychiatry*.

[140] Litman RS, Rosenberg H (2005). Malignant hyperthermia: update on susceptibility testing. *The Journal of the American Medical Association*.

[141] Ochs KL, Zell-Kanter M, Mycyk MB, Toxikon Consortium (2012). Hot, blind and mad: avoidable geriatric anticholinergic delirium. *The American Journal of Emergency Medicine*.

[142] Blackman JA, Patrick PD, Buck ML, Rust RS (2004). Paroxysmal autonomic instability with dystonia after brain injury. *Archives of Neurology*.

[143] Johnson JK, Diehl J, Mendez MF (2005). Frontotemporal lobar degeneration: demographic characteristics of 353 patients. *Archives of Neurology*.

[144] Rosso SM, Kaat LD, Baks T (2003). Frontotemporal dementia in The Netherlands: patient characteristics and prevalence estimates from a population-based study. *Brain*.

[145] Miller BL, Cummings JL, Villanueva-Meyer J (1991). Frontal lobe degeneration: clinical, neuropsychological, and SPECT characteristics. *Neurology*.

[146] Neary D, Snowden JS, Gustafson L (1998). Frontotemporal lobar degeneration: a consensus on clinical diagnostic criteria. *Neurology*.

[147] Rascovsky K, Hodges JR, Knopman D, Mendez MF, Kramer JH (2011). Sensitivity of revised diagnostic criteria for the behavioral variant of frontotemproal dementia. *Brain*.

[148] Coste CP, Sadaghiani S, Friston KJ, Kleinschmidt A (2011). Ongoing brain activity fluctuations directly account for intertrial and indirectly for intersubject variability in Stroop task performance. *Cerebral Cortex*.

[149] Hatanpaa KJ, Blass DM, Pletnikova O (2004). Most cases of dementia with hippocampal sclerosis may represent frontotemporal dementia. *Neurology*.

[150] Neumann M, Tolnay M, Mackenzie IR (2009). The molecular basis of frontotemporal dementia. *Expert Reviews in Molecular Medicine*.

[151] Seeley WW (2008). Selective functional, regional, and neuronal vulnerability in frontotemporal dementia. *Current Opinion in Neurology*.

[152] Lomen-Hoerth C (2004). Characterization of amyotrophic lateral sclerosis and frontotemporal dementia. *Dementia and Geriatric Cognitive Disorders*.

[153] Foulds P, McAuley E, Gibbons L (2008). TDP-43 protein in plasma may index TDP-43 brain pathology in Alzheimer’s disease and frontotemporal lobar degeneration. *Acta Neuropathologica*.

[154] Arai T, Mackenzie IRA, Hasegawa M (2009). Phosphorylated TDP-43 in Alzheimer’s disease and dementia with Lewy bodies. *Acta Neuropathologica*.

[155] Spillantini MG, Yoshida H, Rizzini C (2000). A novel tau mutation (N296N) in familial dementia with swollen achromatic neurons and corticobasal inclusion bodies. *Annals of Neurology*.

[156] Kim EJ, Sidhu M, Gaus SE (2012). Selective fronto insular von Economo neuron and fork cell loss in early behavioral variant of frontotemporal dementia. *Cerebral Cortex*.

[157] Rohrer JD, Guerreiro R, Vandrovcova J (2009). The heritability and genetics of frontotemporal lobar degeneration. *Neurology*.

[158] Hu WT, Wang Z, Lee VMY, Trojanowski JQ, Detre JA, Grossman M (2010). Distinct cerebral perfusion patterns in FTLD and AD. *Neurology*.

[159] Bian H, van Swieten JC, Leight S (2008). CSF biomarkers in frontotemporal lobar degeneration with known pathology. *Neurology*.

[160] Huey ED, Putnam KT, Grafman J (2006). A systematic review of neurotransmitter deficits and treatments in frontotemporal dementia. *Neurology*.

[161] Yener GG, Rosen HJ, Papatriantafyllou J (2010). Frontotemporal degeneration. *Continuum Lifelong Learning in Neurology*.

[162] Mungas D, Jagust WJ, Reed BR (2001). MRI predictors of cognition in subcortical ischemic vascular disease and Alzheimer’s disease. *Neurology*.

[163] Looi JCL, Sachdev PS (1999). Differentiation of vascular dementia from AD on neuropsychological tests. *Neurology*.

[164] Gold G, Giannakopoulos P, Montes-Paixao C (1997). Sensitivity and specificity of newly proposed clinical criteria for possible vascular dementia. *Neurology*.

[165] Ingles JL, Wentzel C, Fisk JD, Rockwood K (2002). Neuropsychological predictors of incident dementia in patients with vascular cognitive impairment, without dementia. *Stroke*.

[166] Varma AR, Laitt R, Lloyd JJ (2002). Diagnostic value of high signal abnormalities on T2 weighted MRI in the differentiation of Alzheimer’s, frontotemporal and vascular dementias. *Acta Neurologica Scandinavica*.

[167] Libon DJ, Price CC, Giovannetti T (2008). Linking MRI hyperintensities with patterns of neuropsychological impairment: evidence for a threshold effect. *Stroke*.

[168] Hauw JJ, Aminoff MJ, Boller F, Swaab DF (2008). The neuropathology of vascular and mixed dementia and vascular cognitive impairment. *Handbook of Clinical Neurology Dementias*.

[169] Delano-Wood L, Abeles N, Sacco JM, Wierenga CE, Horne NR, Bozoki A (2008). Regional white matter pathology in mild cognitive impairment: differential influence of lesion type on neuropsychological functioning. *Stroke*.

[170] Yatsuya H, Folsom AR, Wong TY, Klein R, Klein BEK, Sharrett AR (2010). Retinal microvascular abnormalities and risk of lacunar stroke: atherosclerosis risk in communities study. *Stroke*.

[171] Viswanathan A, Rocca WA, Tzourio C (2009). The Vascular—dementiacontinuum. *Neurology*.

[172] Deschaintre Y, Richard F, Leys D, Pasquier F (2009). Treatment of vascular risk factors is associated with slower decline in Alzheimer disease. *Neurology*.

[173] Becker JT, Huff FJ, Nebes RD, Holland A, Boller F (1988). Neuropsychological function in Alzheimer’s disease. Pattern of impairment and rates of progression. *Archives of Neurology*.

[174] Nebes RD, Brady B (1989). Focused and divided attention in Alzheimer’s disease. *Cortex*.

[175] Baddeley A, Della Sala S, Spinnler H (1991). The two-component hypothesis of memory deficit in Alzheimer’s disease. *Journal of Clinical and Experimental Neuropsychology*.

[176] Collette F, van der Linden M, Delrue G, Salmon E (2002). Frontal hypometabolism does not explain inhibitory dysfunction in Alzheimer disease. *Alzheimer Disease and Associated Disorders*.

[177] Chiaravalloti ND, DeLuca J (2008). Cognitive impairment in multiple sclerosis. *The Lancet Neurology*.

[178] Roca M, Torralva T, Meli F (2008). Cognitive deficits in multiple sclerosis correlate with changes in fronto-subcortical tracts. *Multiple Sclerosis*.

[179] Len TK, Neary JP (2011). Cerebrovascular pathophysiology following mild traumatic brain injury. *Clinical Physiology and Functional Imaging*.

[180] Johnson B, Zhang K, Gay M (2012). Alteration of brain default network in subacute phase of injury in concussed individuals: resting-state fMRI study. *Neuroimage*.

[181] Skuja S, Groma V, Smane L (2012). Alocholism and cellular variability in different brain regions. *Ultrastructural Pathology*.

[182] Sabeti J (2011). Ethanol exposure in early adolescence inhibits intrinsic neuronal plasticity via sigma 1 receptor activation in hippocampal CA1 neurons. *Alcoholism: Clinical and Experimental Research*.

[183] Kazui H (2008). Cognitive impairment in patients with idiopathic normal pressure hydrocephalus. *Brain and Nerve*.

[184] Gleichgerrcht E, Cervio A, Salvat J (2009). Executive function improvement in normal pressure hydrocephalus following shunt surgery. *Behavioural Neurology*.

[185] Tarnaris A, Kitchen ND, Watkins LD (2009). Noninvasive biomarkers in normal pressure hydrocephalus: evidence for the role of neuroimaging—a review. *Journal of Neurosurgery*.

[186] Graus F, Saiz A, Lai M (2008). Neuronal surface antigen antibodies in limbic encephalitis: clinical-immunologic associations. *Neurology*.

[187] Flanagan E, McKeon A, Lennon V (2009). Immunotherapy responsive dementia or encephalopathy: clinical course and predictors of improvements. *Annals of Neurology*.

[188] Rafael H (2005). Secondary Alzheimer started by cryptococcal meningitis (multiple letters). *Journal of Alzheimer’s Disease*.

[189] Ala TA, Doss RC, Sullivan CJ (2004). Reversible dementia: a case of cryptococcal meningitis masquerading as Alzheimer’s disease. *Journal of Alzheimer’s Disease*.

[190] Hoffmann M, Muniz J, Carroll E, de Villasante J (2009). Cryptococcal meningitis misdiagnosed as alzheimer’s disease: complete neurological and cognitive recovery with treatment. *Journal of Alzheimer’s Disease*.

[191] Salvarani C, Brown RD, Calamia KT (2007). Primary central nervous system vasculitis: analysis of 101 patients. *Annals of Neurology*.

[192] Hajj-Ali RA, Singhal AB, Benseler S, Molloy E, Calabrese LH (2011). Primary angiitis of the CNS. *The Lancet Neurology*.

[193] Moore PM, Calabrese LH (1994). Neurologic manifestations of systemic vasculitides. *Seminars in Neurology*.

[194] Foster NL, Heidebrink JL, Clark CM (2007). FDG-PET improves accuracy in distinguishing frontotemporal dementia and Alzheimer’s disease. *Brain*.

[195] Berti V, Pupi A, Mosconi L (2011). PET/CT in diagnosis of dementia. *Annals of the New York Academy of Sciences*.

[196] Migliaccio R, Agosta F, Rascovsky K (2009). Clinical syndromes associated with posterior atrophy: early age at onset AD spectrum. *Neurology*.

[197] Stern Y (2006). Cognitive reserve and Alzheimer disease. *Alzheimer Disease and Associated Disorders*.

[198] Robinson ME, Craggs JG, Price DD, Perlstein WM, Staud R (2011). Gray matter volumes of pain-related brain areas are decreased in fibromyalgia syndrome. *Journal of Pain*.

[199] Obermann M, Nebel K, Schumann C (2009). Gray matter changes related to chronic posttraumatic headache. *Neurology*.

[200] Geha PY, Baliki MN, Harden RN, Bauer WR, Parrish TB, Apkarian AV (2008). The brain in chronic CRPS pain: abnormal gray-white matter interactions in emotional and autonomic regions. *Neuron*.

[201] Apkarian AV, Sosa Y, Sonty S (2004). Chronic back pain is associated with decreased prefrontal and thalamic gray matter density. *Journal of Neuroscience*.

[202] Rainville P, Duncan GH, Price DD, Carrier B, Bushnell MC (1997). Pain affect encoded in human anterior cingulate but not somatosensory cortex. *Science*.

[203] Coghill RC, Sang CN, Maisog JM, Iadarola MJ (1999). Pain intensity processing within the human brain: a bilateral, distributed mechanism. *Journal of Neurophysiology*.

[204] Casey KL (2000). Concepts of pain mechanisms the contribution of functional imaging of the human brain. *Progress in Brain Research*.

[205] Kwan CL, Crawley AP, Mikulis DJ, Davis KD (2000). An fMRI study of the anterior cingulate cortex and surrounding medial wall activations evoked by noxious cutaneous heat and cold stimuli. *Pain*.

[206] Sawamoto N, Honda M, Okada T (2000). Expectation of pain enhances responses to nonpainful somatosensory stimulation in the anterior cingulate cortex and parietal operculum/posterior insula: an event-related functional magnetic resonance imaging study. *Journal of Neuroscience*.

[207] Nielsen FA, Balslev D, Hansen LK (2005). Mining the posterior cingulate: segregation between memory and pain components. *NeuroImage*.

[208] Hu WT, Wang Z, Lee VMY, Trojanowski JQ, Detre JA, Grossman M (2010). Distinct cerebral perfusion patterns in FTLD and AD. *Neurology*.

[209] Kadir A, Darreh-Shori T, Almkvist O, Wall A, Långström B, Nordberg A (2007). Changes in brain 11C-nicotine binding sites in patients with mild Alzheimer’s disease following rivastigmine treatment as assessed by PET. *Psychopharmacology*.

[210] Hilker R, Thomas AV, Klein JC (2005). Dementia in Parkinson disease: functional imaging of cholinergic and dopaminergic pathways. *Neurology*.

[211] Shin SS, Verstynen T, Pathak S (2012). High definition fiber tracking for assessment of neurological deficit of traumatic brain injury finding, visualizing and interpreting small sites of damage. *Journal of Neurosurgery*.

[212] Mesaros S, Rocca MA, Kacar K (2012). Diffusion tensor MRI tractography and cognitive impairment in multiple sclerosis. *Neurology*.

[213] Sarter M, Hasselmo ME, Bruno JP, Givens B (2005). Unraveling the attentional functions of cortical cholinergic inputs: Interactions between signal-driven and cognitive modulation of signal detection. *Brain Research Reviews*.

[214] Sara SJ, Hervé-Minvielle A (1995). Inhibitory influence of frontal cortex on locus coeruleus neurons. *Proceedings of the National Academy of Sciences of the United States of America*.

[215] Amat J, Baratta MV, Paul E, Bland ST, Watkins LR, Maier SF (2005). Medial prefrontal cortex determines how stressor controllability affects behavior and dorsal raphe nucleus. *Nature Neuroscience*.

[216] Cramer SC (2008). Repairing the human brain after stroke: I. Mechanisms of spontaneous recovery. *Annals of Neurology*.

[217] Robbins TW TW, Arnsten AFT (2009). The neuropsychopharmacology of fronto-executive function: monoaminergic modulation. *Annual Review of Neuroscience*.

[218] Yerkes RM, Dodson JD (1908). The relation of strength of stimulus to rapidity of habit formation. *Journal of Comparative Neurology and Psychology*.

[219] Arnsten AFT (2000). Through the looking glass: differential noradenergic modulation of prefrontal cortical function. *Neural Plasticity*.

[220] Floresco SB, Magyar O, Ghods-Sharifi S, Vexelman C, Tse MTL (2006). Multiple dopamine receptor subtypes in the medial prefrontal cortex of the rat regulate set-shifting. *Neuropsychopharmacology*.

[221] Floresco SB, Magyar O (2006). Mesocortical dopamine modulation of executive functions: beyond working memory. *Psychopharmacology*.

[222] Li BM, Mei ZT (1994). Delayed-response deficit induced by local injection of the *α*2-adrenergic antagonist yohimbine into the dorsolateral prefrontal cortex in young adult monkeys. *Behavioral and Neural Biology*.

[223] Wang M, Ramos BP, Paspalas CD (2007). *α*2A-adrenoceptors strengthen working memory networks by inhibiting cAMP-HCN channel signaling in prefrontal cortex. *Cell*.

[224] Seamans JK, Robbins TW, Neve K (2009). Dopamine modulation of the prefrontal cortex and cognition function. *Dopamine Receptors*.

[225] Seamans JK, Durstewitz D, Christie BR, Stevens CF, Sejnowski TJ (2001). Dopamine D1/D5 receptor modulation of excitatory synaptic inputs to layer V prefrontal cortex neurons. *Proceedings of the National Academy of Sciences of the United States of America*.

[226] Aston-Jones G, Cohen JD (2005). An integrative theory of locus coeruleus-norepinephrine function: adaptive gain and optimal performance. *Annual Review of Neuroscience*.

[227] Millar KJ, Mackie S, Clapcote SJ (2007). Disrupted in schizophrenia 1 and phosphodiesterase 4B: towards an understanding of psychiatric illness. *Journal of Physiology*.

[228] Mirnics K, Middleton FA, Stanwood GD, Lewis DA, Levitt P (2001). Disease-specific changes in regulator of G-protein signaling 4 (RGS4) expression in schizophrenia. *Molecular Psychiatry*.

[229] Baum AE, Akula N, Cabanero M (2008). A genome-wide association study implicates diacylglycerol kinase eta (DGKH) and several other genes in the etiology of bipolar disorder. *Molecular Psychiatry*.

[230] Manji HK, Lenox RH (1999). Protein kinase C signaling in the brain: molecular transduction of mood stabilization in the treatment of manic-depressive illness. *Biological Psychiatry*.

[231] Yildiz A, Guleryuz S, Ankerst DP, Öngür D, Renshaw PF (2008). Protein kinase C inhibition in the treatment of mania: a double-blind, placebo-controlled trial of tamoxifen. *Archives of General Psychiatry*.

[232] Fields RB, van Kammen DP, Peters JL (1988). Clonidine improves memory function in schizophrenia independently from change in psychosis. Preliminary findings. *Schizophrenia Research*.

[233] Mair RG, McEntee WJ (1986). Cognitive enhancement in Korsakoff’s psychosis by clonidine: a comparison with L-Dopa and ephedrine. *Psychopharmacology*.

[234] Arnsten AFT, Goldman-Rakic PS (1985). *α*2-adrenergic mechanisms in prefrontal cortex associated with cognitive decline in aged nonhuman primates. *Science*.

[235] Arnsten AFT, Steere JC, Hunt RD (1996). The contribution of *α*2-noradrenergic mechanisms to prefrontal cortical cognitive function: potential significance for attention-deficit hyperactivity disorder. *Archives of General Psychiatry*.

[236] Sahakian BJ, Coull JJ, Hodges JR (1994). Selective enhancement of executive function by idazoxan in a patient with dementia of the frontal lobe type. *Journal of Neurology Neurosurgery and Psychiatry*.

[237] Ljungberg T, Ståhle L, Ungerstedt U (1989). Effects of repeated administration of low doses of apomorphine in three behavioural models in the rat. *Journal of Neural Transmission. Parkinson's Disease and Dementia Section*.

[238] Ljungberg T, Ungerstedt U (1976). Reinstatement of eating by dopamine agonists in aphagic dopamine denervated rats. *Physiology and Behavior*.

[239] Ross ED, Stewart RM (1981). Akinetic mutism from hypothalamic damage: successful treatment with dopamine agonists. *Neurology*.

[240] Alexander MP (1995). Reversal of chronic akinetic mutism after mesencephalic injury with dopaminergic agents.. *Neurology*.

[241] Marin RS, Fogel BS, Hawkins J, Duffy J, Krupp B (1995). Apathy: a treatable syndrome. *Journal of Neuropsychiatry and Clinical Neurosciences*.

[242] Watanabe MD, Martin EM, DeLeon OA, Gaviria M, Pavel DG, Trepashko DW (1995). Successful methylphenidate treatment of apathy after subcortical infarcts. *Journal of Neuropsychiatry and Clinical Neurosciences*.

[243] Barrett K (1991). Treating organic abulia with bromocriptine and lisuride: four case studies. *Journal of Neurology Neurosurgery and Psychiatry*.

[244] Holmes VF, Fernandez F, Levy JK (1989). Psychostimulant response in AIDS-related complex patients. *Journal of Clinical Psychiatry*.

[245] Parks RW, Crockett DJ, Manji HK, Ammann W (1992). Assessment of bromocriptine intervention for the treatment of frontal lobe syndrome: a case study. *Journal of Neuropsychiatry and Clinical Neurosciences*.

[246] Brown GL, Linnoila MI (1990). CSF serotonin metabolite (5-HIAA) studies in depression, impulsivity, and violence. *Journal of Clinical Psychiatry*.

[247] Coccaro EF, Siever LJ, Klar HM (1989). Serotonergic studies in patients with affective and personality disorders. Correlates with suicidal and impulsive aggressive behavior. *Archives of General Psychiatry*.

[248] Coccaro EF (1989). Central serotonin and impulsive aggression. *British Journal of Psychiatry*.

[249] Hollander E, Wong CM (1995). Body dysmorphic disorder, pathological gambling, and sexual compulsions. *Journal of Clinical Psychiatry*.

[250] Olivier B, Mos J (1986). Serenics and aggression. *Stress Medicine*.

[251] Bakchine S, Lacomblez L, Benoit N, Parisot D, Chain F, Lhermitte F (1989). Manic-like state after bilateral orbitofrontal and right temporoparietal injury: efficacy of clonidine. *Neurology*.

[252] Tariot PN, Schneider LS, Cummings J (2011). Chronic divalproex sodium to attenuate agitation and clinical progression of Alzheimer disease. *Archives of General Psychiatry*.

[253] Baxter LR, Clark EC, Iqbal M, Ackerman RF, Lichter EG, Cummings JL (2001). Cortical subcortical system in the mediation of obsessive compulsive disorder. *Frontal Subcortical Circuits in Psychiatric and Neurological Disorders*.

[254] Baxter LR, Schwartz JM, Bergman KS (1992). Caudate glucose metabolic rate changes with both drug and behavior therapy for obsessive-compulsive disorder. *Archives of General Psychiatry*.

[255] Wong DF, Brašić JR, Singer HS (2008). Mechanisms of dopaminergic and serotonergic neurotransmission in Tourette syndrome: clues from an in vivo neurochemistry study with PET. *Neuropsychopharmacology*.

[256] Brody AL, Saxena S, Schwartz JM (1998). FDG-PET predictors of response to behavioral therapy and pharmacotherapy in obsessive compulsive disorder. *Psychiatry Research—Neuroimaging*.

[257] Stern L, Zohar J, Cohen R, Sasson Y (1998). Treatment of severe, drug resistant obsessive compulsive disorder with the 5HT(1D) agonist sumatriptan. *European Neuropsychopharmacology*.

[258] O’Connor K, Todorov C, Robillard S, Borgeat F, Brault M (1999). Cognitive-behaviour therapy and medication in the treatment of obsessive-compulsive disorder: a controlled study. *Canadian Journal of Psychiatry*.

[259] Giacino JT, Whyte J, Bagiella E (2012). Placebo-controlled trial of amantadine for severe traumatic brain injury. .. *The New England Journal of Medicine*.

[260] Willmott C, Ponsford J (2009). Efficacy of methylphenidate in the rehabilitation of attention following traumatic brain injury: a randomised, crossover, double blind, placebo controlled inpatient trial. *Journal of Neurology, Neurosurgery and Psychiatry*.

[261] Huey ED, Putnam KT, Grafman J (2006). A systematic review of neurotransmitter deficits and treatments in frontotemporal dementia. *Neurology*.

[262] Lebert F, Stekke W, Hasenbroekx C, Pasquier F (2004). Frontotemporal dementia: a randomised, controlled trial with trazodone. *Dementia and Geriatric Cognitive Disorders*.

[263] Chollet F, Tardy J, Albucher JF (2011). Fluoxetine for motor recovery after acute ischaemic stroke (FLAME): a randomised placebo-controlled trial. *The Lancet Neurology*.

[264] Hyman SE (2007). Can neuroscience be integrated into the DSM-V?. *Nature Reviews Neuroscience*.

[265] Carlat DJ (2010). *Unhinged: The Trouble with Psychiatry—A Doctor’s Revelations about a Profession in Crisis*.

[266] Rimer J, Dwan K, Lawlor DA (2012). Exercise for depression. *Cochrane Database of Systematic Reviews*.

[267] Lee JC, Blumberger DM, Fitzgerald P, Daskalakis Z, Levinson A (2012). The role of transcranial magnetic stimulation in treatment-resistant depression: a review. *Current Pharmaceutical Design*.

[268] Farahani A, Correll CU (2012). Are antipsychotics or antidepressants needed for psychotic depression: a systematic review and meta-analysis of trials comparing antidepressant or antipsychotic monotherapy with combination treatment. *Journal of Clinical Psychiatry*.

[269] Apostolova I, Block S, Buchert R (2010). Effects of behavioral therapy or pharmacotherapy on brain glucose metabolism in subjects with obsessive-compulsive disorder as assessed by brain FDG PET. *Psychiatry Research: Neuroimaging*.

[270] Butler AC, Chapman JE, Forman EM, Beck AT (2006). The empirical status of cognitive-behavioral therapy: a review of meta-analyses. *Clinical Psychology Review*.

[271] Martin SD, Martin E, Rai SS, Richardson MA, Royall R (2001). Brain blood flow changes in depressed patients treated with interpersonal psychotherapy or venlafaxine hydrochloride: preliminary findings. *Archives of General Psychiatry*.

[272] Dougherty DD, Weiss AP, Cosgrove GR (2003). Cerebral metabolic correlates as potential predictors of response to anterior cingulotomy for treatment of major depression. *Journal of Neurosurgery*.

[273] Chamberlain SR, del Campo N, Dowson J (2007). Atomoxetine improved response inhibition in adults with attention deficit/hyperactivity disorder. *Biological Psychiatry*.

[274] Panitch HS, Thisted RA, Smith RA (2006). Randomized, controlled trial of dextromethorphan/quinidine for pseudobulbar affect in multiple sclerosis. *Annals of Neurology*.

[275] Miller A, Pratt H, Schiffer RB (2011). Pseudobulbar affect: the spectrum of clinical presentations, etiologies and treatments. *Expert Review of Neurotherapeutics*.

[276] Davidson RJ (2012). *Begley S in Their Book the Emotional Life of Your Brain*.

[277] Fava GA, Tomba E (2009). Increasing psychological well-being and resilience by psychotherapeutic methods. *Journal of Personality*.

[278] Hodie;lzel BK, Ott U, Gard T (2008). Investigation of mindfulness meditation practitioners with voxel-based morphometry. *Social Cognitive and Affective Neuroscience*.

[279] Cappa SF, Benke T, Clarke S, Rossi B, Stemmer B, van Heugten CM (2005). EFNS guidelines on cognitive rehabilitation: report of an EFNS task force. *European Journal of Neurology*.

[280] Wolf SL, Winstein CJ, Miller JP (2006). Effect of constraint-induced movement therapy on upper extremity function 3 to 9 months after stroke: the EXCITE randomized clinical trial. *Journal of the American Medical Association*.

[281] Wolf SL, Thompson PA, Winstein CJ (2010). The EXCITE stroke trial: comparing early and delayed constraint-induced movement therapy. *Stroke*.

[282] George MS, Lisanby SH, Avery D (2010). Daily left prefrontal transcranial magnetic stimulation therapy for major depressive disorder: a sham-controlled randomized trial. *Archives of General Psychiatry*.

[283] Ramachandran VS, Altschuler EL (2009). The use of visual feedback, in particular mirror visual feedback, in restoring brain function. *Brain*.

[284] McCabe CS, Haigh RC, Ring EFJ, Halligan PW, Wall PD, Blake DR (2003). A controlled pilot study of the utility of mirror visual feedback in the treatment of complex regional pain syndrome (type 1). *Rheumatology*.

[285] Yavuzer G, Selles R, Sezer N (2008). Mirror therapy improves hand function in subacute stroke a randomized controlled trial. *Archives of Physical Medicine and Rehabilitation*.

[286] Sütbeyaz S, Yavuzer G, Sezer N, Koseoglu BF (2007). Mirror therapy enhances lower-extremity motor recovery and motor functioning after stroke: a randomized controlled trial. *Archives of Physical Medicine and Rehabilitation*.

[287] Franceschini M, Agosti M, Cantagallo A, Sale P, Mancuso M, Buccino G (2010). Mirror neurons: action observation treatment as a tool in stroke rehabilitation. *European journal of physical and rehabilitation medicine*.

[288] Sale P, Franceschini M (2012). Action observation and mirror neuron network a tool for motor stroke rehabilitation. *European Journal of Physical and Rehabilitation Medicine*.

